# Comparative Genomic Analysis and Functional Identification of CER1 and CER3 Homologs in Rice Wax Synthesis

**DOI:** 10.3390/biology15020166

**Published:** 2026-01-16

**Authors:** Nesma E. E. Youssif, Bowen Yang, Haodong Huang, Mohamed Hamdy Amar, Mohamed Ezzat, Mohammad Belal, Sanaa A. M. Zaghlool, Huayan Zhao, Dong Fu, Shiyou Lü

**Affiliations:** 1 State Key Laboratory of Biocatalysis and Enzyme Engineering, School of Life Sciences, Hubei University, Wuhan 430062, China; nesma.eliwa@gmail.com (N.E.E.Y.); bwy@stu.hubu.edu.cn (B.Y.); haodonghuang@hubu.edu.cn (H.H.); huayanzhao@hubu.edu.cn (H.Z.); 2 Hubei Hongshan Laboratory, Wuhan 430070, China; 3 Plant Genome Laboratory, Department of Genetic Resources, Desert Research Center, El-Matareya, Cairo 11753, Egypt; dr_mohamed201110@mails.ucas.ac.cn (M.E.); maabdrc@drc.gov.eg (M.B.); 4 Regional Plant Gene Bank in Sinai, Desert Research Center, Cairo 11753, Egypt; mohamed.amar@wbgcas.cn; 5 State Key Laboratory of Plant Diversity and Specialty Crops, Wuhan Botanical Garden of Chinese Academy of Sciences, Wuhan 430074, China; 6 Agricultural Botany Department, Faculty of Agriculture, Ain Shams University, Cairo 11241, Egypt; sanaa_zaghloul@agr.asu.edu.eg; 7 State Key Laboratory of Woody Oil Resources Utilization, Changsha 410004, China; 8 Yuelushan Laboratory Non-Wood Forests Variety Innovation Center, Changsha 410004, China

**Keywords:** rice, cuticular waxes, alkanes, biosynthesis

## Abstract

Alkane is a predominant wax component, whose production requires the aids of *CER1* and *CER3*. In rice, *OsCER1* and *OsCER3* are present in multiple copies. Until now, the roles of these genes have been studied individually; however, a systematic comparison of their relative contributions to cuticular wax biosynthesis has not yet been carried out. Phylogenetic tree analysis revealed that *CER1s* and *CER3s* from different plants are classified into two subgroups. RT-qPCR analysis showed that these genes display distinct expression patterns, revealing their specific roles in wax production. Promoter prediction analysis showed that cis-elements responding to light, phytohormones and stress are enriched in the promoter region of *OsCER1s* and *OsCER3s*. These proteins are all localized in the endoplasmic reticulum. Further study showed that *OsCER1s* and *OsCER3s* are inclined to form a complex during the wax synthesis. Finally, the wax analysis of single mutants showed that among the examined genes, *OsCER3a* mutation greatly reduced the total wax amounts to 19.6% of wild-type plant with a decrease in most of wax components, whereas mutation of other genes including *OsCER3b*, *OsCER3c*, *OsCER1a* and *OsCER1c* slightly or barely affect wax production, suggesting that OsCER3a plays major roles in rice wax production whereas other proteins redundantly participate in the wax synthesis. Additionally, the wax increasing rates of Arabidopsis expressing *OSCER1* are lower than those of overexpressing *AtCER1*. Taken together, our study identified the predominant genes involved in wax production, which will be useful for genetically engineering rice with enhanced stress tolerance.

## Simple Summary

This study provides a comprehensive characterization of *CER1* and *CER3* gene families in rice, revealing their evolutionary divergence into two distinct subgroups despite originating from a common ancestral lineage. These genes display different tissue-specific expression patterns. Promoter analysis identified an abundance of cis-regulatory elements responsive to light and drought, suggesting potential involvement in abiotic stress adaptation. Subcellular localization confirmed endoplasmic reticulum (ER) targeting, consistent with their enzymatic functions in wax biosynthetic pathways. Protein–protein interaction assays further revealed that OsCER1 proteins interact with OsCER3 homologs, mirroring conserved interactions observed in dicot species such as Arabidopsis thaliana. Functional analysis of transgenic and knockout lines highlighted OsCER3a as a key regulator of wax accumulation, while other homologs exhibited partial or redundant activity. Collectively, these findings enhance our understanding of CER1-CER3 module functionality in rice and offer promising targets for genetic engineering aimed at improving cuticular wax deposition and abiotic stress resilience in cereal crops.

## 1. Introduction

Plant cuticular wax together with cutin forms a hydrophobic layer on plant surfaces against water loss and provides protection from various abiotic and biotic stresses [1,2,3,4]. Cuticular waxes are classified into two types: intracuticular waxes, embedded within the cutin layer, and epicuticular waxes, which form the crystal structure [1,5]. Cuticular wax biosynthesis initiates in epidermal plastids with de novo fatty acid production, followed by transport to the endoplasmic reticulum. There, the fatty acid elongase (FAE) complex progressively extends these precursors into very-long-chain acyl-CoAs (VLCFA-CoAs) through iterative elongation reactions [1]. After elongation, these VLCFA-CoAs are converted into wax components via alcohol-forming and alkane-forming pathways [1]. The alcohol-forming pathway (acyl-reduction pathway) produces primary alcohols and wax esters, while the alkane-forming pathway (decarbonylating pathway) yields alkanes, aldehydes, secondary alcohols and ketones [5,6]. In the alcohol-producing pathway, VLCFA-CoAs are either directly reduced by fatty acyl-CoA reductase (FAR3/CER4) or sequentially converted via a two-step reduction catalyzed by CER3 and SOH1 [7,8]. The primary alcohols are subsequently esterified with C16 fatty acids by wax ester synthase/diacylglycerol transferase (WSD1) to produce wax esters [9]. Alkane production requires the coordinated activity of the CER1, CER3 and CYTB5 (Cytochrome B5), in which CER1 and CER3 play core roles [10,11,12,13,14]. Although CER3 and CER1 have different enzymatic activities, they share similar amino acid sequences and likely evolved from a common ancestor [12,14]. The *CER3* gene encodes a putative VLCFA reductase, which is supposed to catalyze the production of VLCFA aldehydes from VLCFA-CoA, while the *CER1* gene, crucial for very-long-chain (VLC) alkane biosynthesis, encodes a decarbonylase that converts VLC aldehydes into corresponding alkanes [10,11,14,15].

Alkanes play important roles across different plant species. *AtCER1* overexpression significantly increases the odd-numbered alkanes, thus decreasing cuticle permeability and enhancing drought resistance [15]. Similarly, a study performed on tobacco showed that alkanes are positively related to drought and heat resistance [16]. Our recent study also confirms that drought stress induces alkane biosynthesis [8]. Furthermore, under water-limited conditions, CER3 preferentially associates with CER1, thereby channeling very-long-chain fatty acids (VLCFAs) into the alkane-forming pathway [8]. These studies demonstrate that the interaction between CER1 and CER3 is essential for the production of alkanes and the assembly of the efficient sealing layer upon various stresses [3].

In rice, eleven potential homologs of the maize *Glossy1* (*GL1*)/Arabidopsis *CER3*/*WAX2*/*YRE*/*FLP* gene have been identified [17]. These Glossy1-like genes are categorized into two groups based on sequence similarity, i.e., the *CER3*-related group (*OsGL1–1*, *OsGL1–2* and *OsGL1–3*) and the *CER1*-related group (*OsGL1–4*, *OsGL1–5*, *OsGL1–6* and *OsGL1–7*) [18]. To date, although many wax-related genes have been cloned and individually characterized, their functional differentiation within gene families remains unclear, and their roles in certain organs are not clearly defined yet. In addition, most studies have focused on Arabidopsis rather than major crops; the regulatory networks underlying wax biosynthesis in rice, maize and wheat are still poorly defined, creating a bottleneck for crop improvement. This study addresses that gap by using rice as a model to dissect the key factors involved in alkane and primary alcohol biosynthesis. The findings refine the wax biosynthetic network in rice and provide novel targets and strategies for genome editing aimed at enhancing stress resistance, thereby contributing both theoretical insights and practical tools to strengthen food security.

Leaves are the primary organs of photosynthesis, a physiological process essential for plant growth. The wax and cutin layer deposited on the leaf surface provides critical protection against various environmental stresses, ultimately influencing crop yields. To explore which OsCER1 or OsCER3 members play major roles in leaf wax production, we first characterized the physicochemical properties of rice CER1 and CER3 proteins and identified their phylogenetic relationships. We then profiled transcript abundance across organs and stress treatments and scanned the promoter regions for cis-elements that might confer tissue- or stimulus-specific expression. Because our earlier work indicated that CER1 and CER3 assemble into a complex that directs carbon flux toward either alkanes or alcohols, we used the luciferase complementary assay (LCA) to detect interactions between the two gene families. Finally, to compare their roles in wax production, we also examined the wax phenotype of rice or Arabidopsis expressing or knocking out either of these genes. All in all, our study will be helpful to uncover the relationship between CER1 and CER3 and to strictly define their precise roles in wax production. This work provides a valuable genomic and functional resource with potential applications for enhancing stress resistance and productivity in cereal crops.

## 2. Materials and Methods

### 2.1. Plant Materials and Growth Conditions

*Arabidopsis thaliana* (Col-0 ecotype) and rice (*Oryza sativa* subsp. japonica cv. Zhonghua 11) were used in this study. For Arabidopsis, seeds were surface-sterilized and sown on half-strength Murashige and Skoog (½ MS) medium, stratified at 4 °C for 3 days, and then transferred to a growth chamber set at 22 °C under long-day conditions (16 h light/8 h dark). After 10 days, seedlings were transferred to soil-filled pots measuring 7 × 7 × 7.8 cm^3^ and maintained under the same photoperiod and temperature. Rice seeds were imbibed in water at 37 °C for 4 days and then sown in pots with Yoshida nutrient solution supplemented with 47.632 mg L^−1^ Na_2_SiO_3_·9H_2_O. Rice plants were grown in a growth chamber under 14 h light at 30 °C and 10 h dark at 26 °C. Growth conditions for both Arabidopsis and rice were maintained at 65% relative humidity with a light intensity of 200 µmol photons m^−2^ s^−1^. Arabidopsis was irrigated every three days, whereas the nutrient solution for rice was refreshed weekly. Each experimental sample included three biological replicates, with each replicate consisting of 3–4 individual Arabidopsis or rice plants. *OsCER3a* and *OsCER1a* mutants were generated by BioRun Company (Wuhan, China). Single mutants of *oscer3b* and *oscer3c* (T1 generation) were bought from Biogle Genetech (www.biogle.cn).

### 2.2. Methods

#### 2.2.1. Phylogenetic Analysis and Protein Structure Prediction

A phylogenetic analysis was performed to investigate the evolutionary relationships and divergence patterns of CER1 and CER3 homologs across rice species. Genomic sequences from nine representative *Oryza* species were utilized to construct a phylogenetic tree, enabling the assessment of gene conservation and lineage-specific diversification (Table S1); all available CER1 and CER3 query sequences in rice and Arabidopsis were identified through Blastp searches using Arabidopsis CER1 and CER3 protein sequences as the reference genome. Phylogenetic trees were generated with FastTree V2 [19] and Geneious Prime 2025.1.1 [20], employing 1000 bootstrap replicates to assess branch support. *CER1* genes from the same selected *Oryza* species were used as an outgroup, and the tree was constructed using the Jones–Taylor–Thornton (JTT) model with 20 site-rate categories. Model selection was further validated using ProtTest [21], which compared the JTT and Markov models to identify the best-fitting substitution model. Among the available amino acid substitution models, JTT has been the default choice in the majority of plant protein phylogenies (e.g., Arabidopsis, rice, maize) and was therefore adopted here without further model comparison. Evolutionary calibration of the *CER*-based tree was performed with the relative-time maximum likelihood method in MEGA-X [22], converting relative branch lengths (amino acid substitutions) into absolute divergence times by applying external calibration points as temporal constraints. These calibration points provided independent evidence linking genetic changes to real-world timescales. Additionally, the 3D structures of the rice CER1 and CER3 proteins were predicted using Swiss-Model [23]. Model accuracy was assessed using the Global Model Quality Estimation (GMQE) score, and sequence identity was calculated to determine the percentage of identical amino acids between target sequences and selected templates.

#### 2.2.2. Chromosomal Localization and Motif Analysis

The percentage similarity and identity of rice and Arabidopsis protein sequences were calculated with the Ident and Sim online sequence manipulation tool (http://www.bioinformatics.org/sms2/ident_sim.html, accessed on 13 January 2026) [24]. In addition, segmental and tandem duplications were analyzed to determine their contributions to genome evolution. DnaSP v6.12.03 was used to calculate the synonymous (Ks) and non-synonymous (Ka) substitution rates for homologous *CER1/3* gene pairs [25]. Segmentally and tandemly duplicated genome regions were visualized using TBtools 2.390 [26]. Furthermore, the chromosomal positions of *OsCER1* and *OsCER3* genes were identified using the MapGene2Chromosome v2.0 visualization platform (http://mg2c.iask.in/mg2c_v2.0/, accessed on 13 January 2026). Genomic coordinates were obtained from the *Oryza sativa* genome database, and the loci were subsequently mapped onto their respective chromosomes to illustrate the spatial distribution of these genes within the rice genome. Moreover, the identification of conserved motifs in the CER1 and CER3 protein sequences was carried out using the MEME Suite web server (v5.5.0). The analysis was performed in classic motif discovery mode, with the number of repetitions set to “any” and the maximum number of motifs limited to ten. Data including statistical significance scores (E-values), motif lengths, and inter-motif variability metrics for every identified motif are provided in Table S2.

#### 2.2.3. Cis-Acting Element Prediction and Expression Pattern Analysis

For cis-element screening, the 2 kb regions upstream of the candidate genes were analyzed to identify candidate regulatory elements using PlantCARE [27]. For expression pattern analysis of the desired genes in response to various abiotic stresses, the relevant information was downloaded from the public database (https://bar.utoronto.ca/efprice, accessed on 13 January 2026). Drought-, salt- and cold-stress data were obtained from Jain et al. [28]; anoxia data from Lasanthi-Kudahettige et al. [29]; and heat-stress data from Robertson et al. [30]. The heatmap was created by Graphpad Pism9.5 software. The 2 Kb upstream sequences of OSCER1a, OSCER1b, OSCER1c, OSCER3a, OSCER3b and OSCER3C are provided in Supplementary Data S1.

#### 2.2.4. Quantitative RT-PCR (RT-qPCR) Analysis

Total RNA was extracted from plant tissues using the Universal Plant Total RNA Extraction Kit (BioTeke, Beijing, China), including 10-day-old seedlings and other organs at the heading stage. Using 1 μg of total RNA as a template, first-strand cDNA was synthesized using HiScript II First-Strand cDNA Synthesis Kit (Vazyme, Nanjing, China). PCR was carried out on the Bio-Rad CFX96 PCR system (Bio-Rad, Hercules, CA, USA). The thermal cycling conditions consisted of an initial denaturation at 95 °C for 2 min, followed by 39 amplification cycles of 95 °C for 10 s and 60 °C for 30 s. Each experimental condition was analyzed with three biological replicates and three technical replicates. RT-qPCR was performed according to the manufacturer’s protocol (Vazyme, Nanjing, China), with *AtACTIN2* (for Arabidopsis) and *OsUBQ5* (for rice) used as internal references [8,31]. The 2^−ΔΔCT^ method was used to calculate the relative expression levels of each gene in different tissues. The primers used in this study are listed in Table S3. The experiment was conducted using at least three biological replicates.

#### 2.2.5. Plasmid Construction

To generate *OsCER1a* and *OsCER3a* mutants, we constructed expression vectors using the pYLCRISPR::Cas9-pUbi-MH plasmid as the backbone. These vectors contain guide RNA sequences targeting the respective genes: *OsCER1a*: 5′-AATGGCCGTGGCATAGGCTGGGG-3′ and 5′-GCTGCGGGCGGTCTGGTAACGGG-3′; *OsCER3a*: 5′-AGCGGGCGAGCCCCGACCACTGG-3′ and 5′-GGCCCCTGGCGTCCCACAGCGGG-3′.

To investigate the expression of *OsCER1s* or *OsCER3s* in tobacco or Arabidopsis, we cloned the full length of their corresponding coding sequences into the pFGC-eYFP vector using a one-step cloning method (Biomed). To explore the interaction between *OsCER1s* and *OsCER3s*, we cloned the full length of their corresponding coding sequences into pCAMBIA 1300-NLuc or pCAMBIA 1300-CLuc vector using the same method as described above. All plasmids used for vector construction were verified by Sanger sequencing. The pFGC-eYFP vector was constructed as CaMV 35S::CDS::eYFP::OCS terminator. The CAMBIA-based vectors were constructed as CaMV 35S::CDS::nLUC::E9 terminator or CaMV 35S::cLUC::CDS::E9 terminator.

#### 2.2.6. Transient Tobacco Transformation and Production of Arabidopsis Transgenic Plants

*A. tumefaciens* GV3101 cells carrying the recombinant *pEGC-eYFP* plasmid were cultured to prepare a feeder culture. A single colony of *A. tumefaciens* was transferred into 5 mL of LB medium supplemented with kanamycin (50 μg mL^−1^) and rifampicin (50 μg mL^−1^) and incubated at 28 °C with shaking for 24–48 h. This starter culture was subsequently used to inoculate 500 mL of LB medium containing the same antibiotics and incubated at 28 °C for 16–24 h until the culture reached the stationary phase, indicated by an optical density at 600 nm (OD_600_) of 1.6–1.8. Transient transformation of *Nicotiana benthamiana* was conducted as described by Sparke et al. [32]. The target genes, along with an endoplasmic reticulum (ER) marker (CD3-959), were co-transformed into the leaves. After a 2-day incubation period post-infiltration, fluorescence was captured on a Zeiss LSM 980 confocal laser-scanning microscope. eYFP was excited at 514 nm and detected between 535 and 580 nm, whereas mCherry was excited at 561 nm and collected from 590 to 620 nm.

The Arabidopsis plants (Col-0) were transformed by the *Agrobacterium* strain containing *35S:OsCER1a-eYFP* or *35S:AtCER1-eYFP* through the floral-dipping method. The T0 transgenic seeds were evenly sown in the soil. After germination, 1:1000 diluted Basta herbicide was sprayed on the plants to screen for positive transgenic lines. Finally, the confirmed OE lines (OsCER1a OE-4, OsCER1a OE-6, AtCER1 OE-1 and AtCER1 OE-2) were selected for further study.

#### 2.2.7. Luciferase Complementary Assay (LCA)

LCA was carried out according to previous studies [33]. Constructs encoding OsCER1-nLUC and cLUC-OsCER3 were individually transformed into *Agrobacterium tumefaciens* strain GV3101 via the freeze–thaw method. The colonies were selected on the solidified Luria–Bertani (LB) medium, where four bacterial combinations were prepared: empty-nLUC + empty-cLUC, OsCER1-nLUC + empty-cLUC, empty-nLUC + cLUC-OsCER3 and OsCER1-nLUC + cLUC-OsCER3. Each strain was cultured overnight in 5 mL of liquid LB medium supplemented with kanamycin (50 μg/mL), gentamicin (50 μg/mL) and rifampicin (50 μg/mL) at 28 °C and 220 rpm. Subsequently, 1 mL of culture was transferred to 5 mL liquid LB medium containing 1.2 μL acetosyringone (100 mM) and incubated for an additional 4 h. Bacterial cells were harvested by centrifugation at 12,000 rpm for 1 min at room temperature, washed twice with 1 mL osmotic buffer, and cultures were suspended overnight in 1 mL osmotic buffer (half-strength liquid Murashige and Skoog (MS) medium containing 150 μM of acetosyringone, pH 5.7). The solution was then injected into *N. benthamiana* leaves. The optical density at 600 nm (OD_600_) was measured after 20-fold dilution to determine bacterial concentration. Based on OD_600_ values, 80/A μL of each original suspension was diluted to 2 mL with osmotic buffer. For infiltration, 1 mL of each prepared suspension was mixed to generate the four combinations.

Fully expanded leaves of *N. benthamiana* were divided into four regions and infiltrated with the respective bacterial mixtures using a 1 mL needleless syringe, targeting the abaxial surface away from major veins. Plants were maintained in darkness for 24 h post-infiltration and then returned to standard growth conditions. At 72 h post-injection, leaves were sprayed with fluorescein potassium salt (0.2 mg/mL) and kept in the dark for 5–10 min to allow chlorophyll luminescence to decay. Luminescence signals were captured using a Tanon 5200 CCD imaging system (Tanon, Shanghai, China), and relative luciferase activity was quantified as luminescence intensity per unit leaf area (cm^2^). Each experiment was conducted with at least three biological replicates, and representative images from one replicate are shown.

#### 2.2.8. Cuticular Wax Analysis

Waxes were collected from the leaves of 4-week-old Arabidopsis or 3-week-old rice (*Oryza sativa* L.). The cuticular wax composition of leaves from wild-type, different overexpression and mutant lines was examined as described by Lü et al. [34], with slight modifications. Leaf samples were submerged in hexane for 30 sec, and samples with the internal standard n-Tetracosane were evaporated under nitrogen. Waxes were derivatized by incubation in *N*,*O*-bis(trimethylsilyl)trifluoroacetamide (BSTFA) at 100 °C for 15 min. Silylated samples were analyzed on an Agilent 8860 gas chromatograph (GC) equipped with DB-5 (30 m 0.25 mm × 0.25 µm; Agilent, Santa Clara, CA, USA) capillary column and a flame ionization detector, using helium as the carrier gas. The column temperature was initially set at 80 °C and gradually increased at 40 °C min^−1^ to 200 °C, at which point the temperature remained unchanged for 10 min. The temperature was then increased gradually at 3 °C per min and finally reached 320 °C, at which point the temperature was held for 20 min. The quantification was performed based on flame ionization detector (FID) peak areas relative to the internal standard n-Tetracosane. The experiment was conducted using at least three biological replicates, each with three or four technical replicates.

#### 2.2.9. Statistical Analysis

All statistical analyses were performed using one-way analysis of variance (ANOVA) followed by Tukey’s multiple-comparison test (significance threshold *p* < 0.05) to compare samples from the different lines. Figures were generated with GraphPad Prism 9.5.0 (https://www.graphpad.com/scientific-software/prism/, accessed on 13 January 2026).

## 3. Results

### 3.1. Physicochemical Properties and Phylogenetic Analysis of CER1 and CER3 Genes in Rice

A comprehensive analysis identified three copies each of *CER1* (*OsCER1a–c*) and *CER3* (*OsCER3a–c*) in the japonica rice (*Oryza sativa* L.) genome. These genes exhibited distinct physicochemical properties, including variations in genomic length, coding sequences (CDS), protein length, molecular weight (MW) and isoelectric points (*p*I) (Table 1). The genomic lengths of these *OsCER1/3* genes range between 4 kb and 7 kb, and the corresponding CDS is about 1.9 kb in length. The proteins comprise 595–635 amino acids. These proteins are all basic.

**Figure 1:**
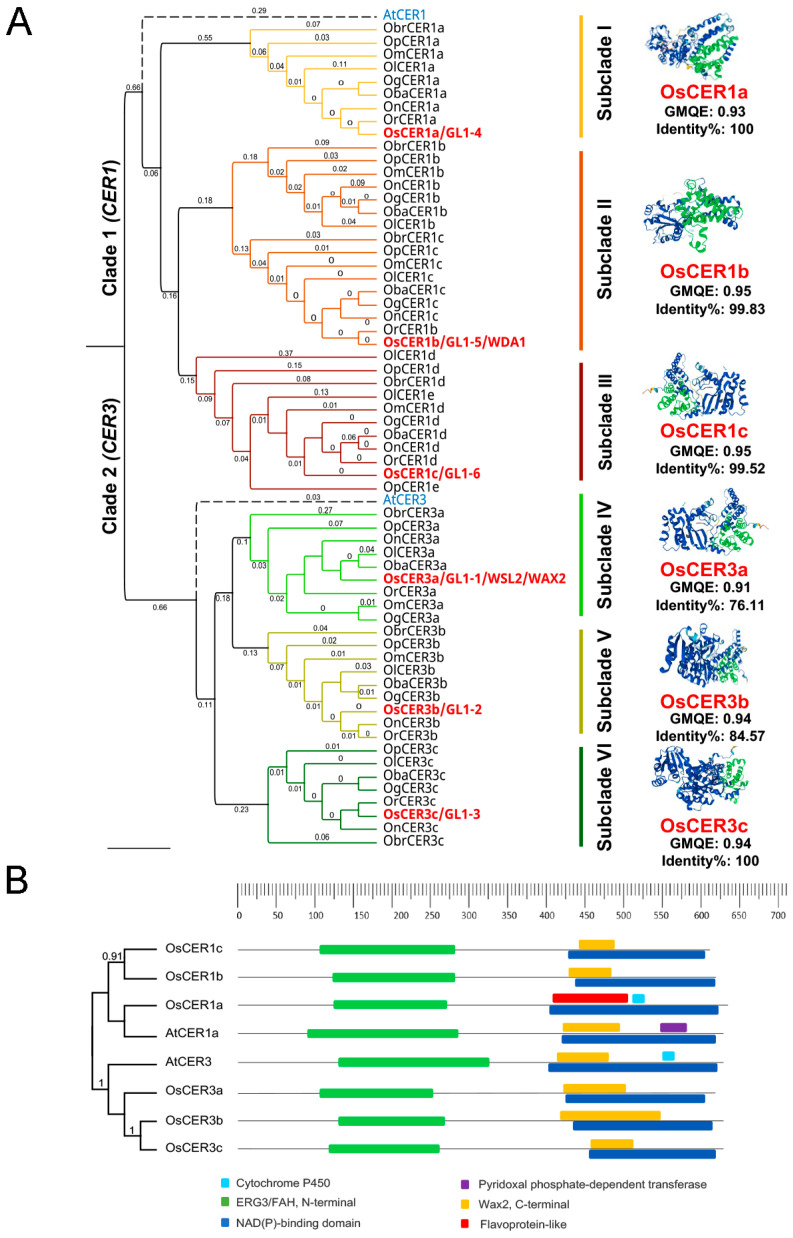
Phylogenetic tree of *CER1* and *CER3* from nine *Oryza* species and functional domains of *OsCER1s* and *OsCER3s*. (**A**) Time-calibrated maximum likelihood phylogenetic tree of transformed branches cladogram with colored clusters, each representing *CER1* and *CER3*. The phylogenetic tree was constructed in Geneious Prime 2025.1.1. using tree support values with 1000 bootstrap replicates. The rice species used for phylogenetic tree analysis include *Oryza balunga* (*Oba*), *Oryza barthii* (*Obr*), *Oryza glumaepatula* (*Og*), *Oryza longistaminata* (*Ol*), *Oryza meridionalis* (*Om*), *Oryza nivara* (*On*), *Oryza punctate* (*Op*), *Oryza rufipogon* (*Or*) and *Oryza sativa* (*Os*). *OsCER1* and *OsCER3* genes are colored red, while Arabidopsis *CER* genes are shown with dotted lines as reference genes. On the right side, the representative crystal structures of the full-length OsCER1 and OsCER3 proteins were displayed. Beneath each 3D model, the GMQE score and sequence identity percentage are provided to indicate the quality metrics used in protein structure prediction. (**B**) Schematic domain architecture of the rice CER proteins showing key domains. Differently colored boxes represent the functional domains, and their positions reflect the same colors in the crystal structures forms.

Phylogenetic analysis of Arabidopsis *CER1* and *CER3* gene homologs across *Oryza* species classified the genes into two distinct clades (Figure 1A): *CER1* (Clade I) and *CER3* (Clade II). Tree supports values were displayed on the tree to show branch reliability, with higher numbers meaning stronger data support for that grouping, using 1000 bootstrap replicates. Each clade is further divided into three well-defined subclades, with each subclade containing a single *OsCER1* or *OsCER3* gene paralog. Notably, the Arabidopsis homolog *AtCER1* served as an outgroup for the *CER1* clade, providing an evolutionary reference point. The analysis uncovered significant evolutionary patterns among *Oryza CER* genes. The *CER1* clade exhibited an unusual divergence pattern, with its members separating within the *Oryza* lineage prior to the A. thaliana speciation event (estimated divergence time: 0.29). This early divergence contrasts with the typical duplication patterns observed in *Oryza CER1* genes and suggests a unique evolutionary history. Similarly, the *CER3* clade showed a parallel pattern of genus-specific divergence preceding A. thaliana speciation (divergence time: 0.27). Three-dimensional structural modeling showed that all six rice CER proteins share high similarity tertiary structures (Figure 1A). In homology-based protein structure prediction, GMQE and sequence identity were applied as key indicators to assess model accuracy and reliability. The analysis yielded robust structural predictions, reflected by high GMQE values and strong sequence identity percentages (Figure 1A). These findings reveal previously unrecognized evolutionary dynamics in the *CER1/3* gene family and provide valuable insights for future studies on plant cuticle evolution.

To elucidate the functional roles of the predicted *CER1* and *CER3* genes, we performed multiple sequence alignments and analyzed conserved domains associated with enzyme catalysis. Consistent with previous studies on Arabidopsis CER1 and CER3 proteins [11,14]. Our analysis revealed critical conserved motifs essential for alkane biosynthesis (Figure 1B). All identified proteins featured an N-terminal ERG3/FAH (fatty acid hydroxylase) domain, characterized by three His-rich motifs (HXXXH and HXXHH), a hallmark of the fatty acid hydroxylase superfamily. The C-terminal region contained the hydrophilic WAX2 domain (~168 aa) (Figure 1B), which is critical for cuticular wax formation [10]. Also, the results showed that all the candidate genes included Nicotinamide adenine dinucleotide (phosphate) (NAD(P))-binding domain. Furthermore, Notably, *OsCER1a* contains additional functional domains, including a flavoprotein-like domain and a Cytochrome P450 region, which may confer specialized catalytic functions distinct from other *CER1* homologs (Figure 1B). These findings underscore the evolutionary conservation of core catalytic residues while highlighting potential functional diversification among rice *CER1/3* genes.

### 3.2. Chromosome Localization and the Conserved Motifs of Rice CER1 and CER3 Genes

**Figure 2:**
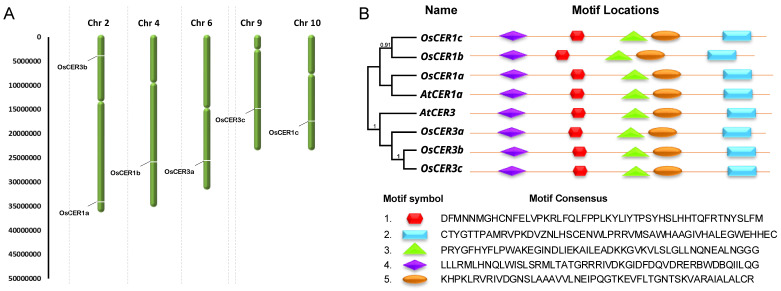
Chromosome localization and the conserved motifs of rice *CER1* and *CER3* genes. (**A**) Distributions of *OsCER1* and *OsCER3* genes on the rice chromosomes. The six predicted rice *CER1,3* genes were mapped on five out of twelve rice chromosomes. (**B**) Phylogenetic analysis and protein structure of *AtCER1*, *AtCER3* and three of rice *CER1s* and *CER3s*. Conserved motifs including fatty acid hydroxylase (purple), acyltransferase (red), cytochrome P450 (green triangle), ABC transporter and lipid transfer protein (LTP) motifs were marked with colored boxes accordingly.

The six identified *CER1/3* genes (three *CER1* and three *CER3* homologs) showed a dispersed chromosomal distribution across the rice genome (Figure 2A). Our mapping analysis revealed their locations on five different chromosomes. *OsCER1a* and *OsCER3b* were co-localized on chromosome 2, while the remaining genes were each located on separate chromosomes (*OsCER1b* on chromosome 4, *OsCER3a* on chromosome 6, *OsCER3c* on chromosome 9 and *OsCER1c* on chromosome 10). This widespread genomic arrangement suggests these genes may have arisen through segmental duplication events followed by chromosomal redistribution during rice genome evolution.

Domain architecture analysis demonstrated remarkable conservation among *CER1/3* proteins (Figure 2B). Both rice and Arabidopsis homologs shared five characteristic protein motifs (fatty acid hydroxylase, Acyltransferase, Cytochrome P450, ABC Transporter and lipid transfer protein (LTP) motifs), with clear separation into *CER1*- and *CER3*-specific groups. The conserved structural features across these dispersed genes indicate strong selective pressure to maintain their biochemical functions despite their genomic redistribution. These findings suggest that while the *CER1/3* gene family has undergone chromosomal dispersion during evolution, the essential protein architecture has been strictly preserved to maintain its role in cuticular wax biosynthesis.

### 3.3. Expression Patterns and Cis-Element Present in the 2 kb Promoter Regions of OsCER1/3s

**Figure 3:**
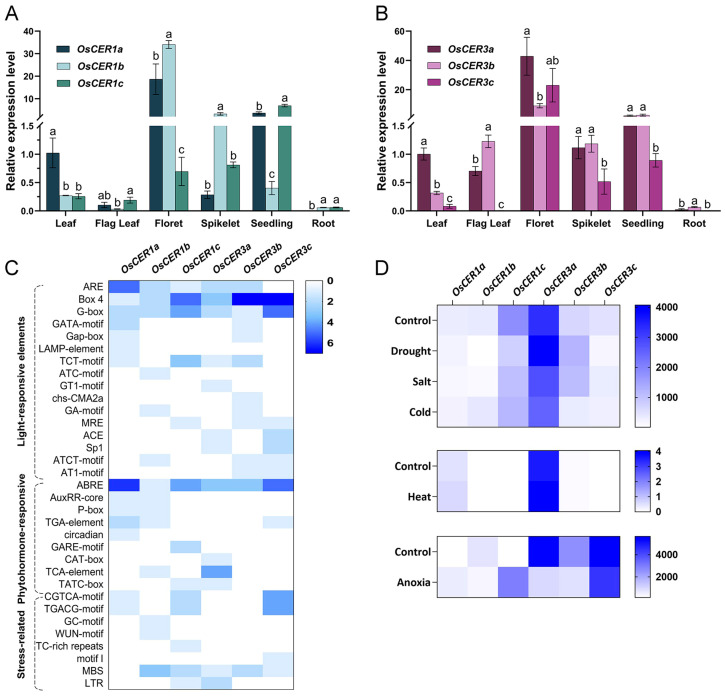
Expression patterns of *OsCER1s* and *OsCER3s* and cis-elements in the 2 kb upstream region of the *OsCER* genes. (**A**,**B**) Organ-specific expression of *OsCER1s* and *OsCER3s*. Each treatment consists of three biological replicates, and all data are shown as means ± standard errors (*n* = 3). Data were statistically analyzed using one-way ANOVA with Tukey’s multiple comparisons test. Different lowercase letters (a, b, c) indicate that the difference is significant (*p* < 0.05). *OsACTIN2* was used as internal control. (**C**) Cis-elements in promoter regions of *OsCER1s* and *OsCER3s*. The color intensity in the cells indicated the repeats of cis-elements in these genes. ARE: anaerobic induction element (matches anoxia-up-regulated genes); Box 4: light-responsive enhancer; G-box: light- and ABA-responsive element as well as a potential heat-responsive motif; GAP-box: light/shade repressor module; TCT-motif: UV-/light-response element; chs-CMA2a: light-inducible chalcone-synthase module; Sp1: light-responsive GC-box; ATC-motif and AT1-motif: auxiliary light-responsive sequences; ABRE: ABA-responsive element; central for drought-inducible expression; AuxRR-core: auxin response; P-box, TATC-box: gibberellin response/repression; circadian: circadian regulation; TGA-element, TCA-element: salicylic acid response; GARE-motif, CGTCA/TGACG-motifs: methyl-jasmonate response; WUN-motif: wound/JA activation; GC-rich repeats: broad-spectrum stress enhancer; MBS: MYB-binding site involved in dehydration responses; LTR: low-temperature responsive element. (**D**) Expression patterns of *OsCER1s* and *OsCER3s* in response to various abiotic stresses based on the published microarray data (https://bar.utoronto.ca/efprice/, accessed on 13 January 2026). The color intensity indicates the expression levels. The raw data underlying the statistical analyses shown in this Figure are provided in Supplementary Table S4.

We analyzed the expression profiles of the *OsCER1* and *OsCER3* genes across various rice tissues (Figure 3A,B). *OsCER1a*, *OsCER1b* and *OsCER1c* exhibit distinct, tissue-specific expression profiles. *OsCER1a* is most abundant in florets, moderately present in seedlings, and low in other organs. *OsCER1b* is also highly floret-enriched, with moderate levels in spikelets. In contrast, *OsCER1c* is only moderately expressed in seedlings and remains low in all other tissues. Though the transcripts of three *OsCER1s* are relatively low in leaves, the expression levels of *OsCER1a* are higher than the other two genes (Figure 3A). *OsCER3* genes, particularly *OsCER3a* and *OsCER3b*, also showed significant expression in floret and seedling tissues, underlining their important roles in wax production of aerial parts. However, as compared with *OsCER3a* and *OsCER3b*, the expression levels of *OsCER3c* are relatively low across different organs, revealing that it might play a minor role in wax production. Similarly to *OsCER1* genes, *OsCER3* genes also have low expression levels in leaves, among which *OsCER3a* expression level is the highest.

Analysis of promoter regions which include 2 kb fragment of the translational start site (ATG) identified 33 cis-acting elements across *OsCER1* and *OsCER3* genes (https://bar.utoronto.ca/efprice/, accessed on 13 January 2026), which were categorized into three major groups including light-responsive, phytohormone-responsive and stress-related elements (Figure 3C). Light-responsive elements dominated the regulatory landscape, with Box 4 showing particularly high representation in *OsCER3c* (eight copies) and *OsCER3b* (five copies), followed by *OsCER1c* (three copies). The G-box, a canonical light-responsive element, occurred in all *OsCER1s* and *OsCER3s* promoters, peaking at four copies in *OsCER3c*. The prevalence of these light motifs parallels the light-induced wax-component synthesis reported in plants [1,35].

Extensive studies have established that cuticular wax biosynthesis is markedly induced by abiotic cues such as heat, drought and salt, with ABA serving as the central mediator of this process [1]. Consistently, ABRE (ABA-responsive element) is the most abundant hormone motif in OsCER1a (four copies), OsCER3c (three copies) and OsCER1c (two copies). MeJA-responsive TGACG/CGTCA elements are restricted to OsCER3c, OsCER1c and OsCER1a; the defense-related TC-rich repeat occurs only in OsCER1c, and the low-temperature motif I is unique to OsCER3c, together implicating coordinated hormonal and stress signaling in the transcriptional control of wax production [1,36].

To test whether the six rice *CER1*- and *CER3*-like genes (*OsCER1a/1b/1c* and *OsCER3a/3b/3c*) possess distinct functions under different abiotic stresses, we inspected their transcript profiles (drought, salt, cold, heat, hypoxia) archived in the eFP-Rice database (https://bar.utoronto.ca/efprice, accessed on 13 January 2026). As shown in Figure 3D, *OsCER1a* is selectively up-regulated by heat and hypoxia; *OsCER1c* is repressed by drought, salt and cold yet induced by anoxia; *OsCER3a* remains high except under anoxia; *OsCER3b* responds positively to drought/salt but negatively to hypoxia; and *OsCER3c* declines under drought and hypoxia. These divergent transcriptional responses indicate that individual homologs may have evolved distinct, condition-specific roles in helping rice adapt to varying environmental cues.

We next compared the promoter cis-element landscape (Figure 3C) with the stress-expression profiles retrieved from eFP-Rice (Figure 3D). A partial, but incomplete, congruence emerges. The heat- and anoxia-induced expression of *OsCER1a* coincides with its G-box and ARE motifs; the drought/salt repression yet anoxia induction of *OsCER1c* aligns with its ABRE and ARE copies; the near-constitutive expression of *OsCER3a*, except under anoxia, corresponds to an ARE-depleted promoter; and the drought/salt up-regulation but hypoxia down-regulation of *OsCER3b* matches an MBS-rich, ARE-lacking architecture. Conversely, *OsCER3c* declines under drought and hypoxia despite carrying both G-box and ARE elements, underscoring that cis-element presence alone is insufficient to predict transcript behavior. These discrepancies—such as *OsCER3b* responding to drought/salt without canonical ABRE/DRE motifs, and *OsCER1a* reacting to heat/hypoxia without evident heat-shock or hypoxia elements—emphasize that promoter-based inferences require experimental validation.

### 3.4. Subcellular Localization of OsCER1s and OsCER3s

**Figure 4:**
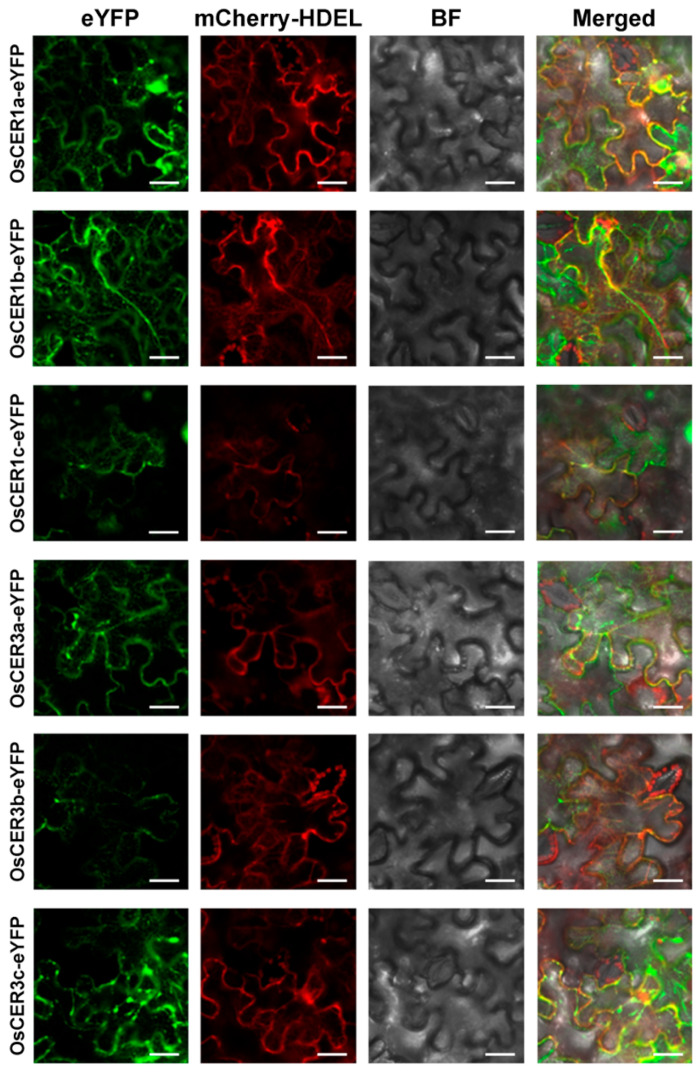
Subcellular localization of OsCER1s and OsCER3s. The fusion constructs were co-transformed with ER marker *mCHERRY-HDEL* into tobacco cells. The signals were detected by confocal microscopy. Bars represent 20 μM. Images shown here are representative of at least three biological replicates. BF stands for bright field.

Previous studies showed that OsCER1s and OsCER3s are located in various compartments including the nucleus, cytoplasm, plasma membrane and/or endoplasmic reticulum (ER) [37,38,39,40,41]. To resolve these conflicting data, each OsCER1/OsCER3 open reading frame was fused in frame with eYFP and co-expressed in *N. benthamiana* with an established ER marker. All fusion signals of these fused proteins co-localized with the ER-marker (Figure 4), demonstrating that every member of the OsCER1 and OsCER3 families is exclusively ER-localized. This result aligns with the well-established fact that cuticular wax biosynthesis occurs on the ER membrane and that the enzymes involved in this pathway are predominantly ER-residing [5,6].

### 3.5. Protein Interactions Detected by LCA

**Figure 5:**
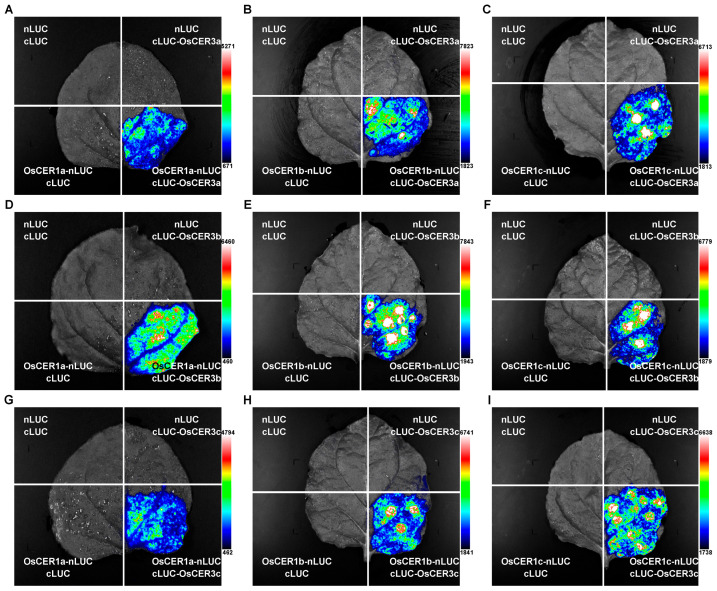
Interactions between OsCER1s and OsCER3s detected by LCA experiments. The different combinations of OsCER1a and OsCER3a (**A**), OsCER1b and OsCER3a (**B**), OsCER1c and OsCER3a (**C**), OsCER1a and OsCER3b (**D**), OsCER1b and OsCER3b (**E**), OsCER1c and OsCER3b (**F**), OsCER1a and OsCER3c (**G**), OsCER1b and OsCER3c (**H**), OsCER1c and OsCER3c (**I**) were co-transformed into tobacco cells. nLUC and cLUC, nLUC and cLUC fused with each *OsCER1a/b/c* genes or *OsCER3a/b/c* genes, and nLUC fused with each *OsCER1a/b/c* genes or *OsCER3a/b/c* genes were used as negative controls. The signals were captured by Tanon 5200 luminescence imaging system. The signal intensity bar at the bottom right shows the range of luminescence intensity. Images shown here are representative of at least three biological replicates.

Previous studies have shown that in Arabidopsis CER1 and CER3 are inclined to form the complex during alkane biosynthesis [10]. To determine whether OsCER1 family members likewise interact with any of the *OsCER3* homologs, we generated constructs in which OsCER1a, OsCER1b or OsCER1c were fused to nLUC, and OsCER3a, OsCER3b or OsCER3c were fused to cLUC, and then monitored pairwise interactions by LCA in *N. benthamiana* leaves (Figure 5). Robust and specific luciferase signals were observed for all pairwise combinations of OsCER1s with OsCER3s, whereas the negative control combinations (nLUC and cLUC, OsCER1s-nLUC and cLUC, and nLUC and cLUC-OsCER3s) produced no detectable luminescence. These results demonstrate that OsCER1a, OsCER1b and OsCER1c each interact with OsCER3 homologs, indicating that, as in Arabidopsis, OsCER1 and OsCER3 proteins act as a complex during wax biosynthesis.

### 3.6. Distinct Roles of OsCER1a and OsCER3s in Shaping Rice Cuticular Wax

To evaluate the roles of *OsCER1a* and *OsCER3s* in rice leaf wax synthesis, we first identified four CRISPR-cas9 mutants by sequencing, including *oscer1a*, *oscer3a*, *oscer3b* and *oscer3c* (Figure S1). *oscer1a* suffered a 1 bp “G” deletion in exon 1, thus shifting the downstream reading frame (Figure S1A). The *oscer3a* mutant was truncated by a 320 bp fragment removal that spans exons 2–3 and eliminates 136 bp after the start codon (Figure S1B); the deletion was further verified by PCR (Figure S1E). In *oscer3b*, a single “A” insertion in exon 3 produced a premature stop, whereas *oscer3c* lost 2 bp (“GC”) from exon 2, once again causing a frameshift (Figure S1C,D). All four mutant lines (oscer1a, oscer3a, oscer3b and oscer3c) were derived directly from the T1 seed that had already segregated away from the Cas9 transgene, and their Cas9-free status was verified by PCR (Figure S2), eliminating any residual editing or off-target activity.

**Figure 6:**
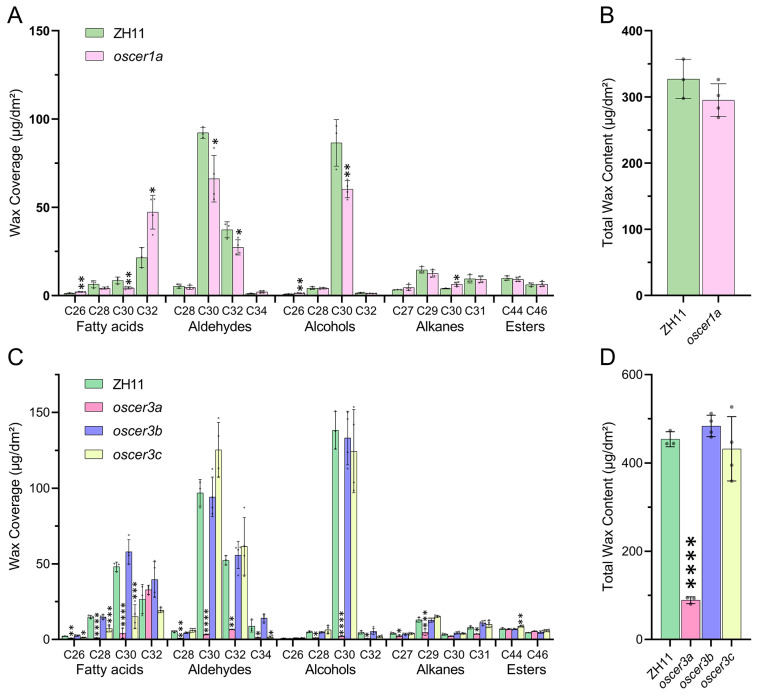
Leaf wax profiles of ZH11 and rice mutants. (**A**) Wax coverage of individual classes (fatty acids, aldehydes, alcohols, alkanes, and esters) in leaves of ZH11 and mutant lines *oscer1a*. (**B**) Total wax content in leaves of ZH11 and mutant lines *oscer1a*. (**C**) Wax coverage of individual classes (fatty acids, aldehydes, alcohols, alkanes, and esters) in leaves of ZH11 and mutant lines (*oscer3a*, *oscer3b* and *oscer3c*). (**D**) Total wax content in leaves of ZH11 and mutant lines (*oscer3a*, *oscer3b* and *oscer3c*). Different mutants are marked with green (ZH11), pink (*oscer3a*), purple (*oscer3b*) and yellow (*oscer3c*) squares. Wax coverage is expressed as wax amounts per leaf/stem surface area (mg·dm^−2^). Each wax constituent is designated by carbon chain length and is labeled by chemical class along the *x*-axis. Values shown are means ± SD (*n* = 3 or 4), which were statistically analyzed using one-way ANOVA followed by Tukey’s multiple comparisons test. * represents *p* < 0.05, ** represents *p* < 0.01, *** represents *p* < 0.001, **** represents *p* < 0.0001. The raw data underlying the statistical analyses shown in this Figure are provided in Supplementary Table S5.

We next surveyed the cuticular wax profiles of the single mutants. Loss of OsCER1a selectively reduced C30 and C32 aldehydes together with C30 primary alcohol while simultaneously elevating C26 and C32 fatty acids; consequently, total wax loads remained statistically unchanged (Figure 6A,B). Among the OsCER3s alleles, *oscer3a* presented the most severe phenotype: nearly every wax class—except esters—collapsed, driving total wax coverage down to only 19.6% of wild-type levels (Figure 6C). In contrast, *oscer3b* plants were virtually indistinguishable from the wild type, indicating that OsCER3b contributes little to wax biosynthesis under our growth conditions (Figure 6C). The *oscer3c* mutant exhibited an intermediate profile: C28 and C30 fatty acids dropped to 26.8% and 18.1%, respectively, while C34 aldehyde fell to 29.4% (Figure 6C). Because these compounds represent minor fractions of the rice wax loads, their reduction translated into no change in overall wax abundance (Figure 6D). These results revealed that in the rice leaf wax production process, *OsCER3a* plays a major role, while *OsCER3b* and *OsCER3c* play minor roles. No visible phenotypic differences were observed among oscer1a, oscer3b, or oscer3c mutants and the wild type, whereas oscer3a plants—owing to a drastic cuticular wax deficit—displayed markedly reduced seed set, indicating that only OsCER3a loss disrupts fertility; this parallels the fertility defects reported for Arabidopsis *cer3* mutants [11].

### 3.7. Ectopic Expression of OsCER1a and Overexpression of AtCER1 in Arabidopsis Differentially Affect Wax Biosynthesis

**Figure 7:**
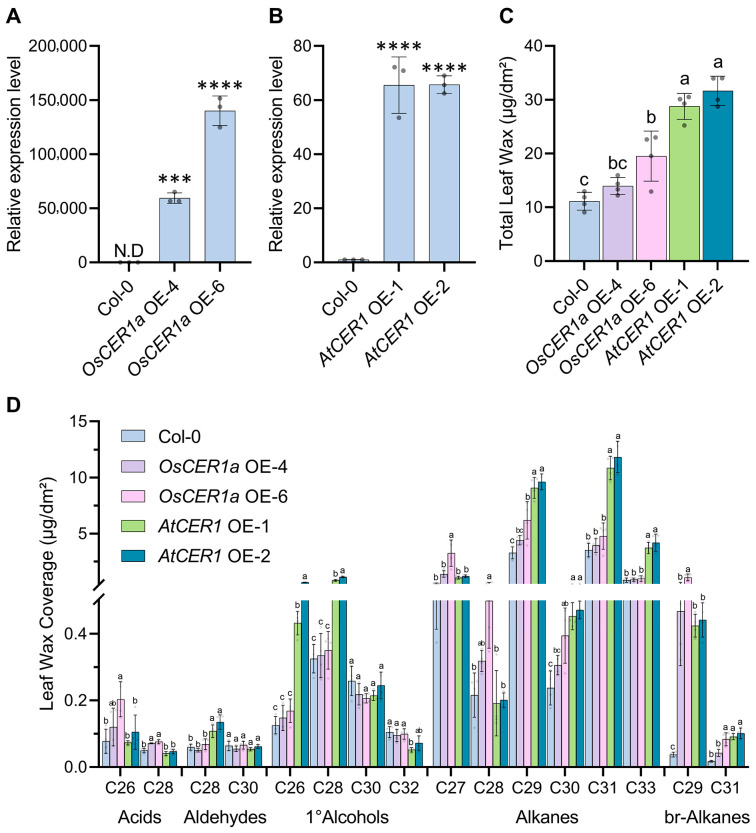
Expression levels of *OsCER1a* and *AtCER1* in Arabidopsis and wax profiles in transgenic plants. (**A**,**B**) Relative expression levels of *OsCER1a* and *AtCER1* in Arabidopsis. *AtACTIN2* gene was used as an internal control. Values shown are means ± SD (*n* = 3 or 4), which were statistically analyzed using one-way ANOVA followed by Tukey’s multiple comparisons test. *** represents *p* < 0.001, **** represents *p* < 0.0001. N.D represents none detectable. (**C**,**D**) Wax determination of Arabidopsis Col-0 transformed with *35S:OsCER1a-eYFP* and *35S:AtCER1-eYFP*. Wax coverage is expressed as wax amounts per leaf/stem surface area (mg·dm^−2^). Each wax constituent is designated by carbon chain length and is labeled by chemical class along the *x*-axis. br-Alkanes stands for branched alkanes. Values shown are means ± SD (*n* = 4), which were statistically analyzed using one-way ANOVA with Tukey’s multiple comparisons test. Different lowercase letters (a, b, c) indicate that the difference is significant (*p* < 0.05). The raw data underlying the statistical analyses shown in this Figure are provided in Supplementary Table S6.

CER1 encodes the rate-limiting alkane-forming enzyme of cuticular wax biosynthesis [8,10,15]. The Arabidopsis cuticle is alkane-dominated, whereas the rice cuticle contains markedly lower alkane abundance. This compositional disparity raises the question of whether AtCER1 and OsCER1 differ in intrinsic catalytic capacity. To address this, *35S:OsCER1a-eYFP* and *35S:AtCER1-eYFP* were individually transformed into Arabidopsis. To verify robust overexpression of *OsCER1a* and *AtCER1* in Arabidopsis, we first performed RT-qPCR on independent T1 lines including OsCER1a OE-4, OsCER1a OE-6, AtCER1 OE-1 and AtCER1 OE-2. RT–qPCR showed a high accumulation of OsCER1a transcripts in the two independent overexpression lines OsCER1a OE-4 and OsCER1a OE-6 (Figure 7A). The expression level of *AtCER1* in AtCER1 OE-1 and AtCER1 OE-2 increased by about 65-fold than in wild-type Col-0 (Figure 7B). All transgenes are C-terminally tagged with eYFP, allowing direct assessment of protein accumulation by confocal microscopy. Strong cytoplasmic eYFP fluorescence was detected exclusively in the four overexpression lines; no signal was observed in untransformed control Col-0 (Figure S3). These data confirmed that both OsCER1a and AtCER1 are successfully transcribed and translated in OE lines. These transgenic plants were subjected to quantitative wax analysis. Total leaf cuticular wax loads are increased in all transgenic OE plants (Figure 7C); however, *35S:AtCER1-eYFP* OE lines accumulated significantly greater wax loads than *35S:OsCER1a-eYFP* OE lines (Figure 7C). The transformed lines carrying *35S:OsCER1a-eYFP* showed modest increases of 25.5% (*OsCER1a* OE-4) and 75.4% (*OsCER1a* OE-6) relative to wild type, whereas *35S:AtCER1-eYFP* OE lines exhibited pronounced elevations of 158.6% (*AtCER1* OE-1) and 184.5% (*AtCER1* OE-2). Detailed profiling revealed that *OsCER1a* overexpression specifically boosted C27 alkane and C29 branched-chain alkane, with only minor changes in other wax fractions. By contrast, *AtCER1* overexpression triggered a broad metabolic shift, markedly increasing C26 and C28 primary alcohols, C27–C31 and C33 n-alkanes, as well as C29 and C31 branched alkanes (Figure 7D). Collectively, ectopic *OsCER1a* overexpression produced only a modest increase in total wax loads, whereas *AtCER1* overexpression resulted in a substantially higher enhancement. These results suggest that OsCER1a may have undergone evolutionary attenuation of its alkane-forming activity; the mechanistic basis for this functional divergence remains to be elucidated.

## 4. Discussion

### 4.1. OsCER3a Emerges as a Prime Target for Precision Engineering of Cuticle Traits in Rice

CER3 was initially identified to be involved in the alkane-forming pathway [10], yet our recent evidence indicates that it additionally governs carbon allocation into both alkane- and alcohol-forming pathways [8]. Rice harbors three CER3 paralogs, but only OsCER3a seems to play dominant roles in wax synthesis. The expression levels of *OsCER3a* are higher than those of *OsCER3b* and *OsCER3c* (Figure 3B,D). Consistent with its expression profile, oscer3a null mutants display a drastic reduction in total cuticular wax (Figure 6). Moreover, we noticed that most of the wax components derived from alcohol- and alkane-forming pathways decreased in the *oscer3a* mutant, confirming its core roles in channeling the carbon resources into alkane- and alcohol-forming pathways in rice as well. Thus, OsCER3a represents an optimal genetic target for engineering wax composition and enhancing stress tolerance in rice.

### 4.2. The Expression Patterns of OsCER1 and OsCER3 Homology Genes Tightly Mirror Their Biological Functions

RT-qPCR results showed that most of the *OsCER1* and *OsCER3* homology genes are highly expressed in florets, whereas they are less expressed in leaves (Figure 3A,B). This is closely related to their biological functions since CER1 and CER3 are reported to form a complex participating in the alkane-forming pathway [8,10,14]. In rice, alkanes play a predominant role in florets but play a subordinate role in leaves (Figure 6) [41,42]. The distribution patterns might be associated with the circumstance where leaves and florets are situated. Rice leaves grow in a persistently moist, hydrophilic microenvironment, whereas the panicle and its florets sit at the aerial apex, exposed to a noticeably drier and hydrophobic atmosphere. Alkanes are nonpolar molecules and thus play dominant roles in floret waxes but only play subordinary roles in leaf waxes. By contrast, the primary alcohols, like polar molecules, exhibit an opposite distribution pattern (Figure 6). The relationship between wax distribution pattern and moisture conditions is not seen in the same plants, which is also seen in plants growing in different conditions. For example, rice waxes are dominated by primary alcohols, while Arabidopsis cultivated in soil contains waxes rich in alkanes (Figure 6 and Figure 7). This phenomenon seems common. One previous study performed in various plants growing in different conditions also demonstrated that the plants growing in arid conditions usually contain high amounts of non-poplar molecules, such as alkanes and esters, whereas those plants residing in high-humidity environments preferentially produce waxes consisting of poplar molecules, including fatty acids and primary alcohols [43]. Collectively, these findings demonstrate that wax deposition is environmentally orchestrated to shield plants from diverse stresses.

### 4.3. The Subcellular Localization of OsCER3a and OsCER3c

Our results showed that OsCER1s and OsCER3s are all localized in ER (Figure 5), among which the localization patterns of OsCER1a, OsCER1c and OsCER3a are consistent with previous studies [38,39,41]. However, the expression pattern of OsCER3c identified in our study is different from that of a previous study, which identified that this protein resides in the plasma membrane [40]. The discrepancy between the two results might result from the usage of different materials. In our study, tobacco cells were used, while onion cells were used instead in the previous study [40]. Moreover, in our study the ER marker was co-transformed with the target genes, which could precisely define the exact sites where the protein resides. However, no marker was used in the previous study, thus possibly causing the misstatement of protein location patterns. In fact, a similar pattern is observed for OsCER3a. One previous study performed in onion cells revealed that this protein is located in various compartments, including the nucleus, cytoplasm, and plasma membrane [37], whereas a later study carried out in rice and Arabidopsis protoplasts that included ER markers clearly placed the protein in the ER. Collectively, these examples underscore that co-expression of appropriate organelle markers is essential to define the exact localization patterns of a certain protein.

### 4.4. Functional Divergence Between OsCER1a and AtCER1 in Arabidopsis Wax Synthesis

*OsCER1a* and *AtCER1* showed high sequence identity (Figure 2), implying functional conservation. However, when expressed in Arabidopsis, their capacities to drive alkane synthesis diverge markedly: *AtCER1* overexpression triggers a substantial wax buildup, whereas *OsCER1a* overexpression exerts only a modest effect. This quantitative disparity suggests that OsCER1a might have undergone evolutionary attenuation of its alkane-forming activity, mirroring the relaxed selective pressure for high alkane output in the monocot lineage. Nevertheless, obtained from a heterologous dicot background, the data may be influenced by several non-mutually exclusive factors that could widen the functional gap; the comparatively low wax accumulation driven by OsCER1a expression may stem from a combination of the following possibilities. First, monocot genes are GC-rich and prefer G/C-ending codons, whereas dicots possess a tRNA isoacceptor pool skewed toward A/T-ending triplets [44,45]. Thus, the transplanted OsCER1a mRNA in a dicot cell might encounter tRNA scarcity, ribosomal pausing, and accelerate mRNA decay, yielding low protein levels. Second, monocots and dicots have evolved distinct enzymatic contexts. For example, the rice hydroxysteroid dehydrogenase OsHSD1 generates primary alcohols [46], and the Arabidopsis AtSOH1 performs an equivalent role [8]; however, our phylogenetic analysis reveals low sequence identity between them. Consequently, although monocot and dicot homologs catalyze the same reaction, each demands its specific partners and catalytic conditions, and any mismatch can affect enzyme activity. These hypotheses await further experimental validation.

## 5. Conclusions

Our study classified *OsCER1* and *OsCER3* into two distinct subgroups, despite their origin from a common ancestor. RT-qPCR analyses revealed that these genes exhibit divergent expression patterns across different organs. Notably, *OsCER1a* and *OsCER3a* showed significantly higher expression levels in leaves compared to their respective paralogs. Subcellular localization via LCA confirmed that both *OsCER1* and *OsCER3* proteins are localized to the endoplasmic reticulum (ER), consistent with their predicted physiological roles in wax biosynthesis. Moreover, protein–protein interaction assays indicated that *OsCER1s* interact with *OsCER3s*, mirroring the conserved interaction observed between *AtCER1* and *AtCER3* in Arabidopsis and suggesting functional conservation of the CER1–CER3 module across dicots and monocots. Phenotypic analysis of transgenic plants overexpressing or lacking *OsCER1* or *OsCER3* genes revealed that *OsCER3a* plays a predominant role in cuticular wax biosynthesis, while the other paralogs contribute to a lesser extent. Collectively, our findings enhance the understanding of the functional divergence and redundancy among *OsCER1* and *OsCER3* genes in rice wax production and identify OsCER3a as a promising genetic target for enhancing cuticular wax formation, improving stress tolerance and supporting breeding strategies aimed at developing stress-resilient rice varieties. Additionally, stress-responsive promoter elements and expression patterns indicate that some homologs, such as *OsCER1c* and *OsCER3c*, may function under conditions like drought or hypoxia. Therefore, future research should define the catalytic roles of OsCER1–OsCER3 combinations and systematically evaluate stress-specific functions of individual homologs under diverse stresses to reveal their condition-specific roles in wax biosynthesis and stress resilience.

## Figures and Tables

**Table 1:** Physicochemical characteristics of *OsCER1s* and *OsCER3s* in japonica rice.

Protein Name	Homologous	Assigned ID	Genomic (bp)	CDS (bp)	Protein (aa)	Mw (kDa)	*p*I	Source of Sequences
OsCER1a	G L1–4	LOC_Os02g40784.1	6074	1860	620	71.52	9.11	NCBI
OsCER1b	GL1–5/WDA1	LOC_Os10g33250.1	5905	1866	595	68.09	7.85	NCBI
OsCER1c	GL1–6	LOC_Os02g56920.1	4319	1908	635	71.64	8.25	NCBI
OsCER3a	GL1–1/WSL2/WAX2	LOC_Os09g25850.1	4527	1860	619	69.66	9.50	NCBI
OsCER3b	GL1–2	LOC_Os02g08230.1	7344	1887	628	71.01	9.73	NCBI
OsCER3c	GL1–3	LOC_Os06g44300.1	7514	1884	627	70.97	9.16	NCBI

## Data Availability

All data supporting the findings of this study are available within the article and its Supplementary Materials. Plasmids, transgenic lines and all other data supporting this study are available from the corresponding author upon request.

## Supplementary Materials

The following supporting information can be downloaded at: https://www.mdpi.com/article/10.3390/biology15020166/s1, Table S1: The detailed information of *CER1* and *CER3* genes among nine *Oryza* genomes; Table S2: Statistical significance scores (E-values), motif lengths and inter-motif variability metrics for every identified motif. Table S3: List of primers used in this study. Table S4: The raw data underlying the statistical analyses shown in Figure 3. Table S5: The raw data underlying the statistical analyses shown in Figure 6. Table S6: The raw data underlying the statistical analyses shown in Figure 7. Figure S1: Molecular identification of different rice mutants generated by the CRISPR/Cas9-based method. Figure S2: Genotyping analysis of Cas9 fragments in different mutants generated by the CRISPR/Cas9-based method. Figure S3: Fluorescent signals of Col-0 and transgenic Arabidopsis lines expressing *OsCER1a-eYFP* or *AtCER1-eYFP*. Supplementary Data S1: The 2 Kb upstream sequences of OSCER1a, OSCER1b, OSCER1c, OSCER3a, OSCER3b and OSCER3C. Ref. [47] is cited in Supplementary Materials.

## Abbreviations

The following abbreviations are used in this manuscript:

LCFA	Long-chain fatty acid
FAE	Fatty acid elongase
ER	Endoplasmic reticulum
VLCFA	Very-long-chain fatty acid
LCA	Luciferase complementary assay
LTP	Lipid transfer protein
