# Supplementary material for: Comparative Genomic Analysis and Functional Identification of CER1 and CER3 Homologs in Rice Wax Synthesis
Source: Biology (Basel). 2026 Jan 16;15(2):166. doi: 10.3390/biology15020166 (PMC12838214; doi:10.3390/biology15020166)
Supplement: Supplementary file 1 [file biology-15-00166-s001.zip › Supplementary Data S1.pdf]

Supplementary Data 1: 2Kb upstream sequences of OSCER1a, OSCER1b, OSCER1c, OSCER3a, OSCER3b and OSCER3C.

Key: 5'UTR CDS

OsCER1a

>O.sativa v7.0|LOC_Os02g40784|Chr2:24718046..24724120
reverse|upstream=2000|downstream=0
GATTAGCAATTTAAAAGATGGGCTAACGAAAGTCGAATAAAATATGTAATTTTAACAGAGATGAACACCGCCAAT
GAGGGATTTTGTACCGGTGGAAAGGAAAATTCTTCCTTTCTCTCTCTCTCTATCTCCGCGGGAAAAATCTCTCGT
GTGCCTTTTCATTATTCTAAGTTTGACTAGCATGATACTAGTAGCAACTAACGCTTCTCTTTTCAACTCCATGGC
ATCACAAGCTGGCTAGTTGCTGCTGGCCAGTGTATCGCCGCGCCGCGAAATAGACGAACCTACAGGGTGTGTTTG
GTTTTGCTTGCACCATTCTAGCCTGGTCCGTATGTTCCATCCAGGCTTACCCTAGCCTGGATAGGTGGGTGCAAA
TAGGGTTGTTAGGTTGGTTGTACGAGTTAAGTTGGTTGATTGTACGCGCAAATTAATCCTGTATACAAGAGATAT
TATTTGGTTTGGTGTATGAGGTTAGAATATGGTTACCTCCTCTTTTGTATGGTAAGATTACCCCTTGCAATTTTA
GTGTACCATTCAATTGTAGTTATAGTACTATTCAATCAGCCAAATTTTGTTAAAATCTGTTGCATATGTAATGGG
TGAATTGGTATAAAGAATTATAAAAAAAATAAATGGTGCCAACGATCAATAATTCGCCGCTTTATATACGCTATA
ACAGGTGAGGGAAAAAAATAAAAAGAAAGAAAAGAAAAAAAATGTGCATCTCGTTCTCGTCGATGTCATCCTCCC
GCCGCCGCACTAATCGTTGCTGCACCGGTCGCCGTCCTCCACCCGCTGCGCTGGTCATCGCCGCAGCCCTCCTCC
GCACCGGTCGTTGTCGTCCTCCACCCGCTATGCTGGCTGCCACCGTCCTCCACTCGCTGCGCTGGTCATCGCCGC
AACCCTCCTCCTCCGCACCGGTCGTCGTCGTCCTCCACCCGCTGCGCCGGTCACTGGTCGTCGCTGTCCTCCACC
CGCTCTGCTGGCTGCCACCGTCCTCCACTCGCTGCGCTGGTCACCGCTGCAGCCCCCTCTCTTTCCCCACTGCTC
GCTACCGGCTACCCTCCCGGATCTGGTGGCCGGCGGCGGAAGAAGGTGCGGGACGGCGACGATTGAAGACGGTGG
CCAGGGATTGAAGAGCTCATCGTCGTTCTCTCCGTCGAGCTCGTCGCCAAACCATCGCCTCTGGCTTCGCTCTCT
GTCTAGGTATGAGGAGGGGGGGGAGAAATGAGGACGGGGCGGTGACTCCATCGCAAGCTGTTGCGTGCTCGGTCC
CGACTCCCTATTAGGAATGCAAGTGGGTAGTCCCGCTATCCGTATAAAAACCCGTTTGCTAGTTTATTTTTCACG
TGGTAGTATAAAATTTAGAAGAAAAAATAAACTAGAAGTGAGATAAGCGGGCTAAAAAAACCCGCTTACCCGCTC
CACTTACATCCCTACTCCTAACACGTCGCCGTGTATAGTTTGTATACATACCCGGGACGAGGCAGGCACCGGGCG
TCGGCAAGCGCGTCCTGCGTGACCGACCGGAAGAAACTTTTTCAGTTGTTGCAACCGCTAGCTCTCGCGCCTTTG
CCTGCCTTGTTGCCTCATGCGCGCGCCGCAACGCAAGAGTGAAACACGGAGGCCGCCGCGCCCGCGTTATGGCGT
AACCGCGCCGCAGTATACCGCATTAAGGTGCTGAGTCATCCCCGTACTGTACCTGTTTCTCTCTCTCTTTTTTTG
ACGCATCAATGCGCCCTCTGCATGCATGCATGCATGCCTGCCTGATTGGAGCCCCTGTCCTTGGCTGCAGTAGTG
ACAGTGGCCACAGACGATGACCTTGTGGCTTGTGCTGATTCTGACCGTCTCGATTGAATGACGACTACTCAAAGA
CACAGACACCCACACCATGAGAGAGGCACAGGAAAGAGAGGCGTGTGCGTCTCCATATGCGCTTCACGCCTCTCT
CACGGCCTCCCTCAACACGGGTATAAAAGGCGAAGCAGTGGGTAGTGCTAGGTTGGGTTGGTGCTTGGCTAGCCA
CGGTATTATTGCGTGAAAGAGGGGGAGGTGAGGACGTGAGGTGAGGTGAGGTGAGGCAGAGTGACCACAGCGACG
GCAGCGTAGCCATGGCGACCAGGCCGGGCCCTTTGACCGAATGGCCGTGGCATAGGCTGGGGAACTTCAAG

OsCER1b

>O.sativa v7.0|LOC_Os10g33250|Chr10:17448950..17454855
reverse|upstream=2000|downstream=0
TACTTTGTCATCAATTCCATTCTGAATGTTCTGGTTGACATTCCGGCGTTCTCGAGCCGTAGTTCTATTTTTTTT
TATTATGTACTCTGGAAACAATCTGTGTCAATAAATGTGTATATATGATTAAAACAGCTAGCAAAATAAAAAATA
AAAAATGCGAAGTGAGCATAGCTCAACTGGTTAGGTTACTTGTGGTGGAACCAGCCCACCCGGGTTCAAATCCTA
GAGTTGACACGGGTGCTCGCATTTACGGCTAATTATTTTTTCAGTGGTAGACGACGTACTCGTCGACAGCGAGGC
ACCTATGGTGACTTCGTCAATCTCAAGACATGCCGGCCCAGTCTTACGGAGGTGCTCATAGGGGTAGGGTGTGCG
TGTGTAATAATAATAATAATAATAATAATAATAATAATAATAATAATAATAATAATAATAATAATAATAATAATA

ATAATAATAATAATAATAAAGAATCAAGAAATACATAGACTTCAAAAGTGGTTTTGGGTAGGAATCTGACTTCCC
GAACTGAGCGTTCGTTTGAAAAACAAAAAGGCACAAAAGTCCCTCGATTTTCACGTGCACACTTTTCAAACTGCT
AAACAGAGAGGCCACCGCTAACTTGAAGAAAGCCCCAAAGAAAGCACTCCACACGTTAACACTCCTGGTCAACTG
GGAAATCTAGAACGAGAGAAACTGTAGAATTTTCCAGCACAAAGAGCTATCTACCGGAAGTCTGTTGGCTAAAAT
TAAAGAGGAGGCCAAAACATGGTCTCGGGCAGGGGCGAGGTTTCCAGCACAAAGAGCTATCTACCGGAAGTCTGT
TGGCTAAAATTAAAGAGGAGGCCAAAACATGGTCTCGGGCAGGGGCGAGGCATCTTGGTAGCTGGCTTGCTTTAT
AATTTTAGCTCATTTGGACCGAAGGTCCTGAGCCTTGTTTTTCTTACTTGTAAAGTTTACTCCTTCTCTATTCAA
TGAAATTGGCAGCTTGCCGATTCGTTAAAAAAACTACTAAACAGTATACATATATACATATATATTTTAAAATTT
TATAAGAATGTTTTAAAAATCATATTAATCTACTTTTTAAAGTTTAAAATAATTAATAATTAATCATACATTAAT
GACTCATCTCTTTTTCTTTTATTCTTAATTTCCCTTTTTTACTCTTCTCAAAGACACAGACTGCCCGAACAGATG
GAGAAGATTGTGTTCCCTTAAGCTCTCCTCTTTGTATTCAAATCAATAATCCACATAAGAAAATATAGCCGGGTC
ACTAGAGCTATTATCTTCAGAAAGACCCAAACCTGATAAAAATCTATCTATTAAGTTTTAGACGATCGATCATAG
CTTTAAACCCCTCGTCGAACACCGGTTTAACGCCGAAACTTGTACTATCATTTTGTTCAGTATCAAAATGGAATT
AATAACCCGTCCTCCTCCCGTAAAGAATTACCTAAAAATACATAATGATCGTATCAATGTTGAAATGCTTTGGTA
GGAAAAAGAAAAACAACATTTTCATGATGGAATTCGATTATATATATATATTTATTATTTGCAGTTAATTATGTT
ACAAATAATAATAAAAAAATAATACAGGAAGGTTAGCTTAGCAGTAACTCGTTTGTTTTTGGTACATTCATGCCG
TCTGCTGCATCTATCACAAACCTGACCCTAGTTACCTATTTCTGACGCACATGATTCATAAAGGACCGATATCTT
CCCTTCGAGCAACCCCAATCAATGCAAGAATATTTATTTTGAAGGTAGAATTATTCAGAAGTTACTGGCATTCTC
TCTGGTACTCCTTCCGTAAAAAAAAAAAAAGCAAATTTAGTTTGATGGGATATATTACAGTGCAACGAAATCTGA
ACATACTTCTCATCTACTCTATCCGTTCTAAAAAAACTTAAATCCGGATTTAAAGTACTATGATGTGCTATATTC
AACTAAAATTCCTTATATTTTAAGATGGGGGAGCACTATGTAAGAGTAGTATATGAGACTCTGACACGTACGCAC
AGCTAGATCGAAGCCCTGGTCGAGTTTATAAATTGAGATATATCCGTCCTCCATTCTCAGGCCAGTTCTTGCAAG
ACTCTTGCCGTGTTGAGACTGCAGAAACGAGAGCTAGCTAGCCAGAGGCTGCAAGAAGATTCTGCACCCATAGAT
CGTCTACCATGGCCACAAACCCAGGACTCTTCACGGAGTGGCCATGGAAGAAGCTTGGCAGCTTCAAG

OsCER1c

>O.sativa v7.0|LOC_Os02g56920|Chr2:34889554..34893873
reverse|upstream=2000|downstream=0
AACGACGTTATACATTAAAATACGGAGGGAGTATATATTTGTTTTGAAAATTTTCTTTACGATTAAATGTAAAAC
GCACATGCACACAACTACTACTCCCACGCGGCCACGCGCCCGCCTACTCGTGAGCACGGATACAACTCGGGAATT
TCATGTTAATGGTTTGGAGATACAATCTGTGTATGCGTAAACATGTGTGTTGAGGCCAGCTGGTACGAATCGTTA
TTCTTTAGGGTTTCATCTTCATATATACCTTTTGTAAGTCACTGTTCGGCGTTATAACCTCACCTTAATATAGTG
AAGATATTTTGCTGGCTAGTGCCCTTGGTTTTTTTCTCTTCTGTTTTAGGTGGGTTTTCCACGTTAAATCTCATA
TCTTTGTGATTGATCTAGTGGTTTTTATCGTTTATTTGCTTATCGTTTCTTAAGAACGTGCGTGTGCTCAAGATT
ATTCAAACTCTTAAATGGTTTTTCGCTTTTATTATGAACTTACAATTATCGATATGTTTTAGCTATCAAAAATGG
GAGAGAAAAGTGTCTACGTACTAGTCACACAAATACGGCAAACGGATTTGTTGGTGGAGCAATGGTCCTAGGCAT
GGATGCTCGCAATCAAGTCTGCTGGCAGGAGGCAGAGAGAGCAACTCTCAGCCACAGGTAGGAGTAGCTCTCATC
GCTAGCAGACACGTACAGAAAAGCACTGTAATTCCAGCTCGAATTCCCCTTCAATGCGCTCAAGATGTGTACCGG
CCGTCCGCAGAATAGACCGGAGCATGCATGCTGTAGCTTTCAACATCCCAACCACCTTCGACCTACTACGACTGC
TATGTACACCCGACCACTACGTACAAATATACTCCCTACTTCCCTAAATATTTGACGTCGTTAATTTTTTTAAAA
ATATTTGACCGTTCGTCTTATTCAAAAACTTTTGTGAAATGTGTAAAACTATATGTATAAATAAAAGTATATTTA
ACAATAAATCAAATGATAGGAAAAGAATTAACAATTACTTACATTTTTTGAATAAGACAAACGGTCAAAGATTTT
TAAAAAAGTCAACGGCGTTAAACATTTTGAGATGGAGGTAGTATTAGTTAGCATCAAAAAGGGAACGTCGTGGTC
TCTCAGGGTACATCGATTTGCCCTTTCAATACGATAACCATGCATGTTGTTATCGCCTCATCCGTGAGTATGGGA
CTACTCCTAACGGCTAATACAAACCTAGGGTGTGTTTGGTTCACGATCATATATGGATGAGATATGCTCATCCAT
ATTTTGTTGAATATAGTGATTTAATATTTTTTTTTTCATTTGGTTTGTACGCATATGATGATTCAACTTTTTGTT
TGGGTGGAACAATGTAGCATGGATAGGAGAAGCCAAAATTTAGTAGGACCCATTTATTATATATACTCTCTATGT

TTCATGTGATAAGATGTTTGACTTTTTTCGTAGACAAATTTTGTTAAGTTTGATAAAGTTTATAGAAAAATATAG
CAACATCTAAAATATTAAATTAGTTTTATTAAATCTAACATTGAATATATTTTGATAATATGTTTTAGATAATAT
TAAATTAGTTAAGAGTTCTCTTCTGCAGTCATCTAAAAAGAGTTCTTCTACAGGTAATTAATTTGGTCTATAGTT
CTATATACGCATGTAGGCAATATGGGAGAACAAAATGGCAATGTATAACAATATATGAGAATAGAATATAAAACA
CTTAATTTTTATTTTTATCTTCATCTCCGGAGGATTCAAACACCACCTAACTGATACTGTTATAGTAAGAGATTT
TAGATGGACCCGCATGCGGACTCCTATTTTAAGCTCATGATTTTCTAAAAAATAAAATATCCAATCAAAATATCA
ACCAGCGTCGCTGGGTTGGACGACAGGAGTAAAAGCTAGCTATCGTCTTTTGATCCGGCCCTGATCAATGGATTT
CCACTCTCTCAGCTCGGCCTATATAAAGCTAACGAGTACACTCCACTCCCACAGCCATCTCCTCTCTACTGTCAA
TCTCAAGTGCGTGCCCGGTATCAGCTCGACCGGTCTCGCACCTGCAACCAAACCACACACAGCTAGCTATCATGG
CCTCTAAGCCAGGGCCTCTCACTCAGTGGCCGTGGCATAACCTTGGAAACTACAAG

OsCER3a

>O.sativa v7.0|LOC_Os09g25850|Chr9:15492997..15497524
forward|upstream=2000|downstream=0
AAATGAATTAGTATGATATTTTAGAGCAACTTTCATATATAAAGTTTTCGCACGAGACGTACCGTTTAGCAGTTT
GAAAAGCATGTCACGAGTATCCAAAATTTAATCTTGTGCTTGCAGAAGAAACGAAGAGGGCCTAACCCATATAAC
CTGCGTCATTAATCTATAGCTTAGCAAAGTTTACTTTTTTGCCTGCCTCCTGGCACAGGAACGCCGACGAAGAAA
AACCTACAGGCTTTCTCAACTATGATTCCTTCCTCAACCTGTGAACTCTGGCGACAGGAGGACGACAGGGGTGTA
TGTGGAGCAGATAATGCACAGTAATAATCCAATCCTTAGGACTTACAAACTGCTGGAATGAACTGACCAAGTACG
TTTAGAACGTAGGAATTTTGACTTATACATGCATAGTGTGATTTGTACGTGAAATACATTAATTAATTTTCTGAA
TTTGTTTCCAATTTCTCGTCGTCAGGCAATGCCGCAATGCCTTGTCTTAAAGATTGCAAATTGAATGCGCCATCT
CCATCGATCTCTCTATCTCCATTGATGGCTGCCCAAAAAATAAAAAATAAAAAGAGCCGACAATGTTTAAGGAGA
GAAGCATACGCGCACGTTTCGTGATGAAACATACATGCAAGACTAATTTCGAATTATATATCACATATTCGCTCT
CTGAATGAGCGTATCGGGTATTTGGAATTTCGTTTGATATGTAAAAGTTGAATTTTAGGTTACTTTTTTGTGGAA
TAATATATCATAAATTAATATTTTTTATAACTATTTATGTGACATGTAAATGACGAGCTGATATCAGTTTAGGAT
AATTTCCTCGCTAGGTGATTGAACGAACCCTGCACGCGGCAGACGTCGTGGCTACTACTATCTAGCTAGCTAGCG
TTGGTTACGCGTGGCTACTATCCAGCCAGAATTGGTTACGCCGTCGCCCATCGATCCGCCACACCACGCCAAACG
CGAACTTAATTGCTACTATCCGATAGTATGTACGCGTACTACACAAAAGTGGGGAAAAGAAGGTTGTCCTGACTC
CATGAGAAGACGATCCAAAACACGGGGCCGGACAACCAAACAAGCTAGCTAGCGGCCGGCGGCGAGCCACCGTCG
TCGTTGCTGAAGACGTCGACTAATTTGGCCTCCCGGCCGGCGCCATCGATCATCTCAAAATTAATACTCCCTCTG
TATAAGGGATTTTGAGTTTTTGTTTGCACTGTTTGATCACTCGTTTTATTAAAAAAATTTGTGACAATATAAAAA
ACGAGAAGTTGTGCTTAAAATACTTCGGATAATAAAGTAAGTAAAAAATAAATAATAATTCTAAAAAAAATTTAA
TAAAATAAGTGTGCAAACAATACAAGCAAAAACTCAAAATCACTTATAATCATGGGACGGAGGGAGTATATAGTA
TCCAAAAATCATCTCAAAATTAAAACCGAATCGAAACTCGCCGGGCCGCGGCCCGCCCGGCCGTCCTGGCCCAAT
TAATTCCGGCGACCGCTGCATGCAACGCTCCGTACGTGATCGCCCAGCGGCTCGCGCGCGCACCACGCGTCCACG
CGTCTCTCTCCTTTATACATGGACAATGGATTAATTTACTTGTAGAAAAAAAAACACGAGGCTAGCTTAGCTTGT
GCCCGGCCCAGCCGCGTGCGTGATTTCCCCAAGTTCACAACCTGTACGTGTGTGTGGTACGTAGCGGTCGCAATC
CCGTTTCAAATCTCGATCGTGTGTGGTGCCATTAACTCCGTCTGGTTGGGGAAGCCGGTGGCGGATAGCCCGACT
GACAGGTGCTGTGGCCTGGCGGCCGGCCGGCCAATCGATGGATGGACGGATGGATCCCATCGATGATCCTTCGTT
GATTCGCGTCAACCTCTCGCTGCCCGAATCGGACTCTCACCTTCCCCGCCCTCCACCGCTGTGACCGCGCGCTGT
CTGTCCCTTGGCTAATACCAGTCGCAGTGCAACTGTGCAAGTGTTTCACGTATATAAAAGCATCCCGAGACGCAG
CGCGCATGACCAGCCGCGTTGGGTGCAAAACCACAACCGCCAACATCTTCTAACTCCCCTCACGGCTCTCCCTCT
CCCCGCACGCACAACACCGGTGAACCCCTACGTACTACTGAGCTGAGCTGAGAGGACGAAGAAGAAGATCAAGCT
CGAGGGGCTTGCGATCATGGGTGCCGCATTCTTGTCGTCGTGGCCATGGGATAACCTCGGCGCGTACAAG

OsCER3b

>O.sativa v7.0|LOC_Os02g08230|Chr2:4355044..4362388
reverse|upstream=2000|downstream=0
ACAAACAGTCTAGCTTTTAGTCCAGATTCTGAGAAGCTGTAGTTGTAGAATTTAGAAAATGAACTAGAAGCCAGA
AACTGGGAAACCCGGCTTTTCCAGATTCTAAGAAGCTGACTACCAACCAGTTGCTTCTTAAAATCTTAAGCTCCC
CCAAACAGGCCCATAGTATCCTCATTTTTTCACCTATATTTATACTTATTATTGATCAAAATTTAAATTTTTAAG
CTTAAATCTGAAGTTAATTTTGAAATTTTTATCCTTATTTTTAGCCTTTGCTTTCAGATCGTTAAGAACATTGTA
TATAAAAGTTTTATTCATAAATTATTTTTCATTTGCAAATATGTCTTTTTACTTTTTCCTCCAATACTTCAACAA
TAAGCTTTATGAGAAAAATAACATGGCTATGCATTAATAGTGTCCATATGTATATGGCTATTATTTTAGTTTCAT
AATTATTTACGTTTTAATATAATCTTACATGCCAATACATGATTAATTGAGGTAACTATTTCTATTTTACCCGTA
TTAAATCAACCAAAATATCATACCATACGGACCATTAATGAAGTTGAATAGTCATGCACTACAGTACTCAAAGCA
TTGATATTGAAAACAACAATACCATTAAGATAGTACTACTCCCTGTGGTTTTATTTATTTGACATTCATTAGTTC
ATTTTTAAACTACTAACAAATTGCTCCACAGGGAAGGAGAGAGTAATTAACTACACAGGGGCAAATTCGACATAA
AACCTTCTCAGAAATGTTCAAACGCCAGGAATTATATATCATCCACAAAACACATTTGCTATAGCTATATTATTC
ATTTTGGGACTTTGGGACACTTTGGGTGTACTATATATTAAATTGGTTATAAATAACGTGACAAATGTTAGTTAT
AGCTATCAGTTATCTATACCATCGAAGTTACTCTTGATCTGTGTACTGGTACTACTAGTATTATGTTGTCAAGAA
CAGGAGGAGAGAAGTATACTCCATCTGCATTTAGTAGCATTGTCTGGCCGGCGCGGCGCAGGTGTCAAAATCTCC
CCTAGCTGGTGGTGTGTGTGCATCGGTTTGGCATGCACTTACTAGTTAATGTTTGTGTTTATGGACAGTTAGTTT
GCAGTTGCAAAATGGCACATACCGTTCTCAGAGTTGGTCCTCAAAAAGAGGAAAAATGATATTAATGGGAGTAAC
ACATGTACTCCTATCTATCGTCTACCTGATATGCAAACACGCGTTTTAATTTGCATGTTTCCATATTGTAAGAAT
TTAACGCAATGGACATGGTCAATTATAGCACAGTAATATTTCTAGCTGGACCGGTATACCATTTGCATCTGCCAT
TACCTACTTGCTCCGACTAGTATAGTAGAAGATATACTAACATTCATCATGAATCATGTATTCAATCCTTTTTTA
AAGATAGTGGATAATAATTATACACAGTCAAAATATAAATACCTCTTTGGTTTAGATTATTAAGTGAATTATTGA
TCCAGATTATTCACTTGATTACAGAGATAAACTAAATATTTTAAGAAAAAAGTAAACTAGGCAAATAGTTAAAAA
TGACTGGTCTAAAGCAATAGGAGTACGATAGAATATTTTTTTTAAAAAAAAGGGACGTAATTTTCTAAGCGTAAA
ACAAATGATTCATTAAAAAAACCCGATGAATAAGCTGGAAATTAAGACCACGGCGCGGTAGATCAATAGATGCAC
CAAATCAGTTTCTTGTACAACCGTTCGGTCTATTACGCAATAGGAGTATATATCTGGTCGTATTTTCCGGAGGAT
ATAGTCATTAATTAATTAGTCCATATATATATAGTACATATTTTATCATCATATTGTTTTAATTAATAATCTAGT
TAATTAGCCGTCTCCGGAACAAATACCCTAATTACTGTACCTAGGTGGGGGCCAACCTAATATGGCGCATGTAAG
TTTCGTCGTCGTCTCCTACACGCGCGTCGTCCCCTTCTTCTCCTTCTCTCGGATCTCTTCTTATAAGTAGAGAGG
GAGACGCAGCGTGTGGCATCCAAAGCTCTCTCTCTCTCTCTCTCATAGCTAGCTAGAGCATGCAGCTACTAGCTA
GCAAGCTTAATTAGCAGCCACACATTAGAGTCACAAAGTGAGAGAGAAGGAGGAGCTTCACAAGCTCGATCGAGA
GAGCAAACTAAGCAAGCAGCCATGGCTGCTCCTCCTCTCTCGTCGTGGCCATGGGCAAGCCTCGGCTCGTACAAG

OsCER3c

>O.sativa v7.0|LOC_Os06g44300|Chr6:26736602..26744116
forward|upstream=2000|downstream=0
TGCACTTCCATTGAGAATCTATAAAATTTCAGTACAACACTGTACATTCTAGTGTTTTTAAGTGAGAAATTAGGA
ATTTGGGTGTTCATGTGTACCCCCAAACACAATATAGCTTCGCCACTGAGTACCGTACATACGTCTCGTAGCTAC
TCTCTCAATTTCCATATTATAAAATAATAGTATTTTTAACACAAAATAAACATATTATCAAAATATATTCAATGT
TAGATTTCATTAAACTAATGTGGTGTTTCAGATGTTCCTATTTTTTTTTATAAATTTAATCAAACTTTTAAAAAAT
TGATTAAAAAACCAAAACCGACTTAATTATAATATGAAAAAGGAGGGAATAACCCGGGTTTACGATTCCGACGAA
ATCCGGTCGGATTTCGTGAAATTTCGACGTTTCGACGAGGCTTGAAAATAGAAACCGTCTGAGATTTCGAGGAGT
TTCATCCAAATTCAAATTTGAATTGATAAAAAAGGAAAAAAATCAAATAAAATCTTATAAATACAATGATATTCA
TAGAACTTGTTGGGATATAAATTTTCGAAAAAAAGATGTATTGTGTGTATTTACTGAAACTTACATCAGGAAAGA
AAAGAATTTCAGCAAATTACCTCTGTTTCAAATTACTGATCGAAATTTCGATGATTTCGATCGAAATTTCTCTAA
AACCGATCGGTTTTCAAGAACTTATCGGCAAACTCGGGGAATTTGTATTCAGATTTTGGTTTATAAAATTTGTTC
GGAATTTATCGGTTTTCCACCGGATTTCGCGAAATTCTGAATTTCGCTAGTGCCCGAAAAGGAAGGGCCTGTTGG

AACCGTAAACGCTGGGAATAACTATTTTTGCTAGACAATAACTGCTACATTCTACTGCTAGTAGCTATGGTAGAA
TTTTTTGGCCATGGCTCTCGTAGTGGTACCGCCCGGCCAAATGCTACTGGTACGTACTGTGGTACTCCCGCAGTT
GCACATCAATTCAATTAGTCACATTGATATGAACAACATATTATATCTGGCCAGTAGTTTTAATTAATAGTACGT
AAAAAGATGCATGCATGTCATCCTAAAATACTATATATATCCGTTCGTAAAAAGATGCATGTCCTCCTAAAATAC
ATGAAATCAAAACGTAAAATATTGAATTGTTAATTGATGCAAAAATAATAGGACTTAAAAATTGAAAATTCTGTC
AGTAGATTTTCCATGAAAAACTCTTTTATATAGCTATAATTTAATATTTTATAAATATATATGTATAGTTTGACA
AATTAATGGTAAACGTGTGCATTAGGGACGACTACTACCGATGTCCAAAACATCATACTTTTGAGAAGGGATGTA
ATTTGTCCATTGTGAAATATAAGGCACTTTTTTTAACTCTAAACTATAACCTTTTTCTTTCTTTTCGTTAGCACA
TTTTTAAAACAATTATACAATGTATTTTTAAGATCTTTAAGTTTATTTTTTTTTTTGTAAAATATATACTTTTAAA
GTTTTGAATAATTAATATTTAATTATTTATGAGCTAATGGATTACCTATAAAAAATATACTCGGTATTTTAGTAA
TTCAACTGTCCGTAAAAAAAATGTTGATGTACTGTTAACCTTTTCAAGTACATGCATGCATGCACGCTCGGCCAG
AGTACGTATTACTGTTTATTGAAAGAAAAAAACATTCAATATGAGAGAAGGACTCTCATATCGAAACTTATTAAA
AGAGAAGTGAGGCTCGAACTTAAATCGACTAGCCCACATCTTACGGTATTAGCCGAAAGACCGGGTCTTTCTACG
TATTGCCATTTCTGTGACTACGTCTTTTTGTTTTTTTAAGAAGAAGAAAAATAAGAAGAGGAGGAGTAGTGAATT
AAGGCAGCGCTCGAGCTGGCTAACCACTGCCAGAGTTGGAAAATAGGAAGGTTGCCATTGAATTCTCTGATGGAC
TTTTCGCTCCTCCCTCTTGTTATAAATAGGGGAGGGTCTTGGCGTCCCAAGAATCATAGGTGGGTGACAGAGAGA
GAGAGATCTCTCCATTCTTCCCAACCCAGCAGTTCTAGCTAGCTAGCTATCTAGTTCATCATCCCCATCCACCGA
GCTCGATATCCCTGGCCATGGCCATCTCCATGGCCTCTCCCTTGTCTTCTTGGCCTTGGGCATTCTTAGGCTCAT
ACAAG
