# Supplementary material for: Comparative Genomic Analysis and Functional Identification of CER1 and CER3 Homologs in Rice Wax Synthesis
Source: Biology (Basel). 2026 Jan 16;15(2):166. doi: 10.3390/biology15020166 (PMC12838214; doi:10.3390/biology15020166)
Supplement: Supplementary file 1 [file biology-15-00166-s001.zip › Supplementary Figures and Tables.pdf]

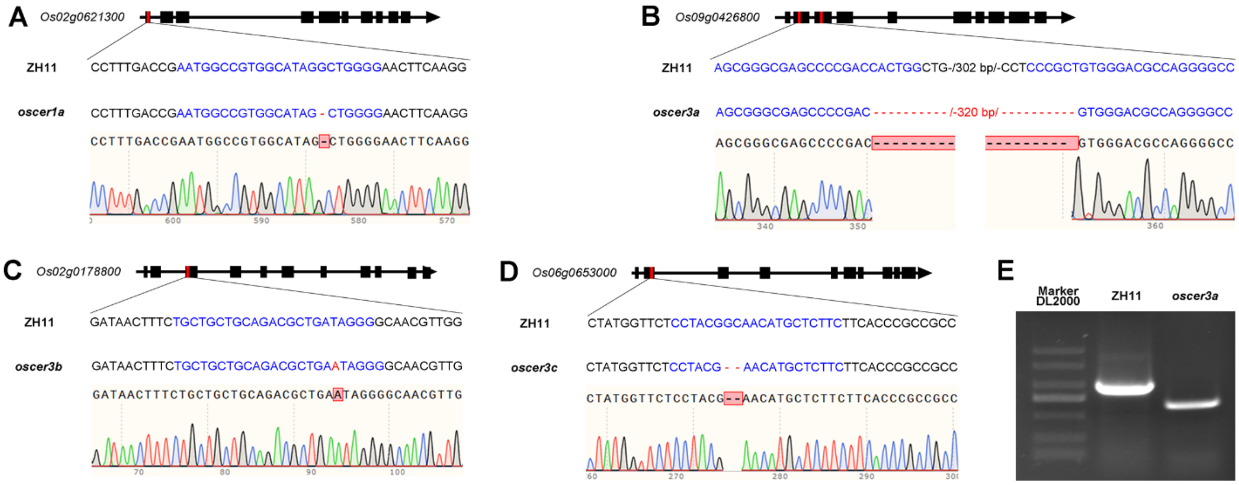

Figure S1. Molecular identification of different rice mutants generated by the CRISPR/Cas9-based method. (A-D), Sequence results show the mutation sites in *OsCER1a* (A), *OsCER3a* (B), *OsCER3b* (C) and *OsCER3c* (D) genes generated by the CRISPR/Cas9-based method. (E) The large deletion of *oscer3a* was further verified by PCR.

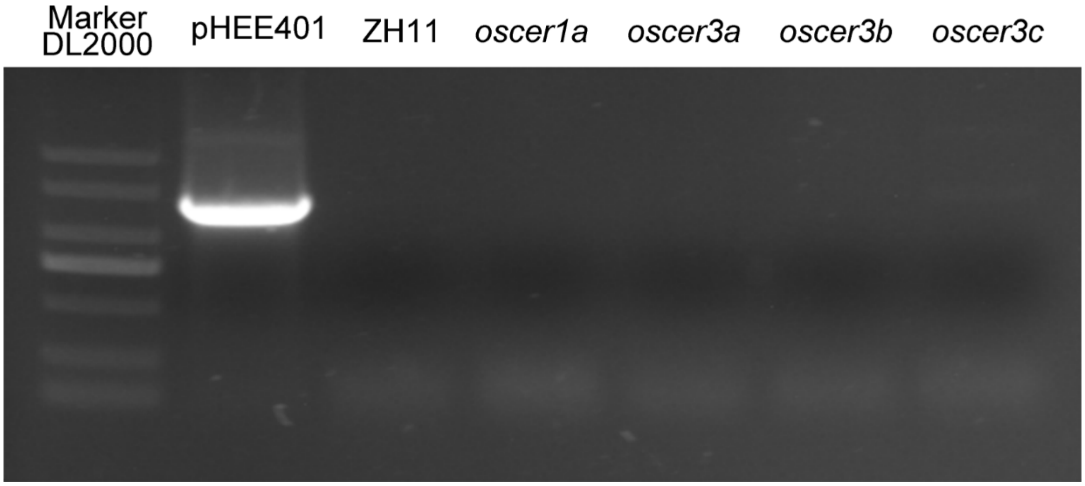

Figure S2. Genotyping analysis of Cas9 fragments in different mutants generated by the CRISPR/Cas9-based method. The pHEE401 plasmid was used as a positive control and the rice wild type ZH11 was used as a negative control.

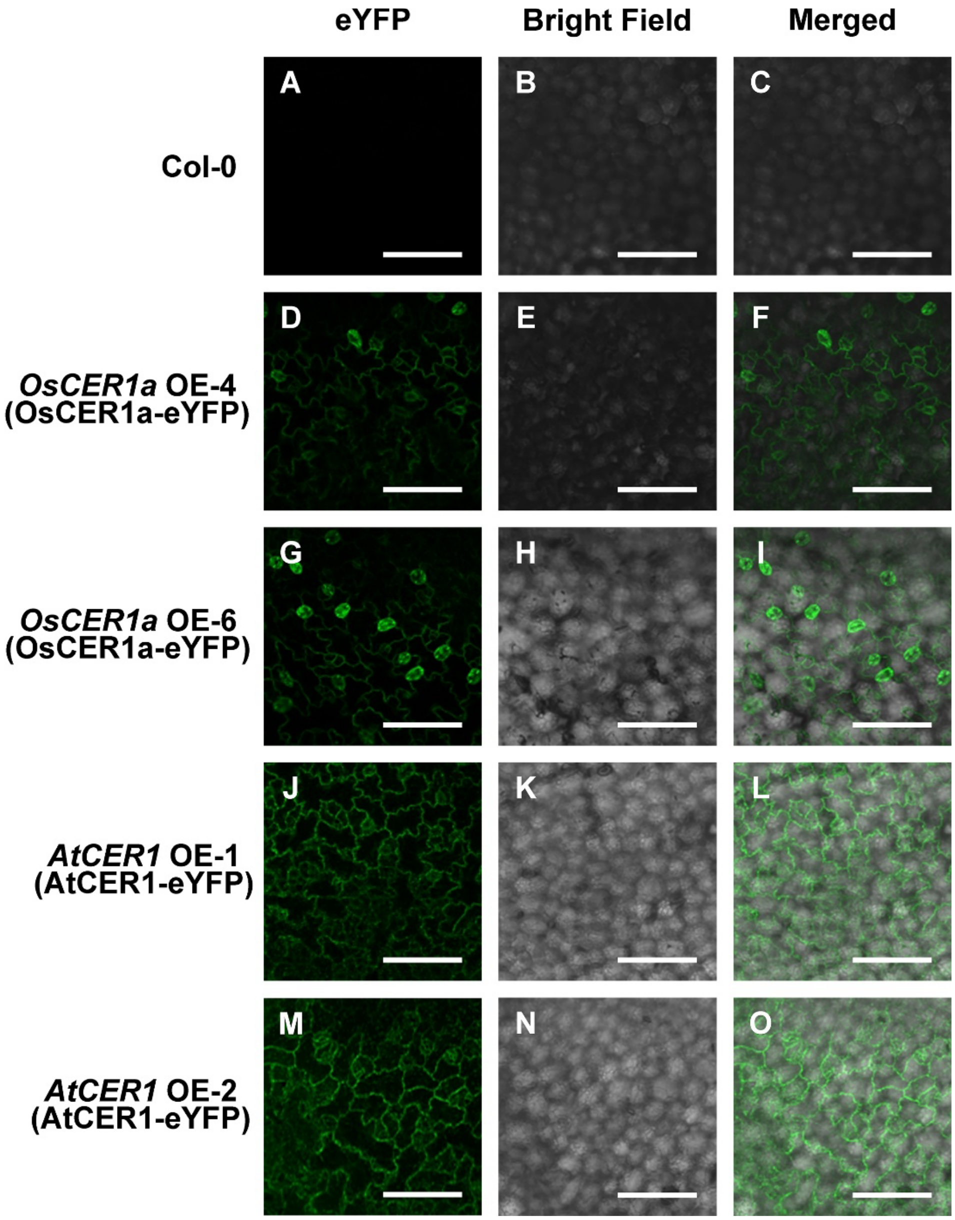

Figure S3. Fluorescent signals of Col-0 and transgenic Arabidopsis lines expressing *OsCER1a-eYFP* or *AtCER1-eYFP*. (A-C) Wild type Col-0; (D-I) Two transgenic Arabidopsis lines expressing *OsCER1a-eYFP*; (J-O) Two transgenic Arabidopsis lines expressing *AtCER1-eYFP*. Bars represent 100 μm.

Table S1. The detailed information of CER1 and CER3 genes among nine Oryza genomes.

ID	Gene identifier	aa	MW (kDa)	isoelectric point (*p*I))
AT1G02205 (AtCER1)	AtCER1	630	73.02358268	7.965015411
AT5G57800 (AtCER3)	AtCER3	632	72.28885108	8.258052826
KN538697.1_FGP046	OlCER1e	659	75.76079958	8.170574188
KN538881.1_FGP046	OlCER3c	586	65.94178678	9.32718277
KN538891.1_FGP013	OlCER1b	673	77.50116188	9.399280548
KN538903.1_FGP015	OlCER1c	416	47.95873548	7.84394455
KN539418.1_FGP009	OlCER3b	516	58.57872858	9.84356308
KN539764.1_FGP003	OlCER1a	1358	152.9039675	6.788166046
KN541830.1_FGP001	OlCER3a	372	42.92204328	7.669574738

KN541966.1_FGP002	OlCER1d	543	62.29955388	8.615871429
LOC_Os02g08230.1	OsCER3b	628	71.01503348	9.726604462
LOC_Os02g56920.1	OsCER1a	635	71.63922268	8.24641037
LOC_Os04g43270.1	OsCER1b	619	71.52312378	9.115856171
LOC_Os06g44300.1	OsCER3a	627	70.97168348	9.160823822
LOC_Os09g25850.1	OsCER3c	619	69.65727548	9.496425629
LOC_Os10g33250.1	OsCER1c	595	68.08578418	7.849658966
OB02G15250.1	ObrCER3b	619	70.46009108	9.26897049
OB02G31830.1	ObrCER1c	621	71.55404008	8.835475922
OB02G43700.1	ObrCER1a	633	71.56507368	8.607486725
OB04G25600.1	ObrCER1b	617	71.22472798	8.647808075
OB06G30860.1	ObrCER3a	625	70.43639188	8.587032318
OB09G17620.1	ObrCER3c	632	70.80571008	9.315380096
OB10G20160.1	ObrCER1d	635	72.73324398	8.250843048
OBART02G05980.1	ObaCER3b	622	70.66574248	9.672504425
OBART02G24440.1	ObaCER1c	619	71.53912328	9.115856171
OBART02G37060.1	ObaCER1a	602	67.79975228	8.434719086
OBART04G18500.1	ObaCER1b	557	64.33470998	8.684658051
OBART06G23140.1	ObaCER3a	627	70.99973728	9.160823822
OBART09G10930.1	ObaCER3c	619	69.69326528	9.496425629
OBART10G12880.1	ObaCER1d	720	82.83082968	8.383556366
OMERI02G06880.1	OmCER3b	667	75.14999608	9.795871735
OMERI02G23970.1	OmCER1c	619	71.45098618	9.115749359
OMERI02G34870.1	OmCER1a	635	71.68928548	8.247264862
OMERI04G15550.1	OmCER1b	662	76.53601858	8.96931076
OMERI06G23070.1	OmCER3a	217	24.16392708	8.388309479
OMERI10G09710.1	OmCER1d	621	71.34951088	8.019275665
ONIVA02G06970.1	OnCER3b	639	72.30050358	9.84532547
ONIVA02G39800.1	OnCER1a	602	67.68461778	8.437549591
ONIVA04G16850.2	OnCER1b	472	53.51515148	8.251163483
ONIVA06G27320.1	OnCER3a	627	70.97168348	9.160823822
ONIVA09G10640.1	OnCER3c	619	69.65727548	9.496425629
ONIVA09G12600.1	OnCER1c	619	71.50909698	9.115856171
ONIVA10G13050.1	OnCER1d	720	82.82885778	8.495548248
OPUNC02G05240.1	OpCER3b	623	70.57549048	9.698246002
OPUNC02G22380.1	OpCER1c	619	71.29883278	8.992542267
OPUNC02G34210.1	OpCER1a	634	71.49222798	7.97121048
OPUNC04G16230.1	OpCER1b	491	57.42681058	7.927845001
OPUNC06G20930.1	OpCER3a	625	70.71835388	9.025814056
OPUNC09G09460.1	OpCER3c	619	69.71538298	9.486225128
OPUNC10G11230.1	OpCER1d	567	64.72634188	8.970058441
OPUNC10G11250.1	OpCER1e	606	69.62241358	7.190151215
ORGLA02G0055300.1	OgCER3b	628	71.05607558	9.670688629
ORGLA02G0211800.1	OgCER1c	619	71.53912328	9.115856171
ORGLA02G0322700.1	OgCER1a	635	71.72433088	8.245342255
ORGLA04G0156100.1	OgCER1b	559	64.46274918	9.077671051
ORGLA06G0193700.1	OgCER3a	627	70.93766628	9.160823822
ORGLA09G0081300.1	OgCER3c	619	69.69326528	9.496425629
ORGLA10G0114600.1	OgCER1d	621	71.20721428	8.022480011
ORUFI02G06110.1	OrCER3b	639	72.31255788	9.84532547
ORUFI02G25810.1	OrCER1b	639	73.74250288	9.123653412
ORUFI02G38530.1	OrCER1a	602	67.71464408	8.437549591
ORUFI06G24800.1	OrCER3a	627	70.97168348	9.160823822
ORUFI09G11730.1	OrCER3c	619	69.65727548	9.496425629
ORUFI10G13590.1	OrCER1c	699	79.97839138	7.349033356

Table S3. List of primers used in this study.

Name	Sequence	Purpose	Reference
qOsUBQ5-F	ACCACTTCGACCGCCACTACT	RT-qPCR	Jain M et al., 2006
qOsUBQ5-R	ACGCCTAAGCCTGCTGGTT		
qAtACTIN2-F	GCACCCTGTTCTTCTTACCGA	RT-qPCR	Li et al., 2025
qAtACTIN2-R	CTTGGATGGCGACATACATAGC		
qAtCER1-F	AAGGATGGGAAATGCATGAG	RT-qPCR	Li et al., 2025
qAtCER1-R	TGATGTGGAAGGAGGAGAGG		
qOsCER1a-F	ACACCCCATCGTTCCATTCT	RT-qPCR	Lu et al., 2025
qOsCER1a-R	AGGTCAGGTGTTTCTTCCGT		
qOsCER1b-F	ACATCCGTCATCCATCCGTT	RT-qPCR	
qOsCER1b-R	GAAGCAGTTCCAGTCAAGGC		
qOsCER1c-F	CAACGACTGCGTCTACCACTG	RT-qPCR	
qOsCER1c-R	CACGGCCATCATGGGTGATG		
qOsCER3a-F	CGTACCACACGATCCATCAC	RT-qPCR	
qOsCER3a-R	TCTTCTGCATCTCCCAGGAC		
qOsCER3b-F	GCGAAGAAAGGCATCAACGA	RT-qPCR	
qOsCER3b-R	TCCATTCAGTGCCTCGTTCT		
qOsCER3c-F	TCTCTTTGATGCCCTGGGAG	RT-qPCR	
qOsCER3c-R	CACCAGGAACACAAAGTCCG		
oscer1a-cas9F	GTTGGTGCTTGGCTAGCCAC	Detecting mutation sites generated by CRISPR/Cas9 editing on *OsCER1a*	
oscer1a-cas9R	AACCCATTGAACAGGATCTGGTC		
oscer3a-cas9F	TGGGTGCAAAACCACAACCG	Detecting mutation sites generated by CRISPR/Cas9 editing on *OsCER3a*	
oscer3a-cas9R	CAGCACCAGCTGCTCCAG		
oscer3b-cas9F	CGGATATCCACGGCAACACT	Detecting mutation sites generated by CRISPR/Cas9 editing on *OsCER3b*	
oscer3b-cas9R	GACCAACACATGCAGCAGC		
oscer3c-cas9F	TCAACTGCTGCTTCAATTCTTTGT	Detecting mutation sites generated by CRISPR/Cas9 editing on *OsCER3c*	
oscer3c-cas9R	CGGTACCTTTTAGATAGCGC		
CAS9-F	GGCTCACGCCCAACTTCA	Detecting the presence of *Cas9* in the genome	
CAS9-R	TCGTCTTCCCGGACTGCT		

1. Jain, M.; Nijhawan, A.; Tyagi, A.K.; Khurana, J.P. Validation of housekeeping genes as internal control for studying gene expression in rice by quantitative real-time PCR. *Biochem. Biophys. Res. Commun.* **2006**, 345, 646-51.
2. Li, S.; Zhang, X.; Huang, H.; Yin, M.; Jenks, M.A.; Kosma, D.K.; Yang, P.; Yang, X.; Zhao, H.; Lü, S. Deciphering the core shunt mechanism in Arabidopsis cuticular wax biosynthesis and its role in plant environmental adaptation. *Nat. Plants* **2025**, 11, 165-175.
3. Lu, H.P.; Liu, X.H.; Wang, M.J.; Zhu, Q.Y.; Lyu, Y.S.; Xu, J.H.; Liu, J.X. The NAT1-bHLH110-CER1/CER1L module regulates heat stress tolerance in rice. *Nat. Genet.* **2025** 57, 427-440.
